# Progress and Challenges of Ferrite Matrix Microwave Absorption Materials

**DOI:** 10.3390/ma17102315

**Published:** 2024-05-14

**Authors:** Xianfeng Meng, Wenlong Xu, Xujing Ren, Maiyong Zhu

**Affiliations:** School of Materials Science and Engineering, Jiangsu University, Zhenjiang 212013, China; 2212205017@stmail.ujs.edu.cn (W.X.); 3220708112@stmail.ujs.edu.cn (X.R.)

**Keywords:** ferrite, microstructure, interface polarization, electromagnetic microwave absorption

## Abstract

Intelligent devices, when subjected to multiple interactions, tend to generate electromagnetic pollution, which can disrupt the normal functioning of electronic components. Ferrite, which acts as a microwave-absorbing material (*MAM*), offers a promising strategy to overcome this issue. To further enhance the microwave absorption properties of ferrite *MAM*, numerous works have been conducted, including ion doping and combining with other materials. Notably, the microstructure is also key factor that affects the microwave absorption properties of ferrite-based *MAM*. Thus, this article provides a comprehensive overview of research progress on the influence of the microstructure on ferrite-based *MAM*. *MAMs* with sheet and layered structures are also current important research directions. For core-shell structure composites, the solid core-shell structure, hollow core-shell structure, yolk-eggshell structure, and non-spherical core-shell structure are introduced. For porous composites, the biomass porous structure and other porous structures are presented. Finally, the development trends are summarized, and prospects for the structure design and preparation of high-performance *MAMs* are predicted.

## 1. Introduction

With the advancement of radar and semiconductor technology, unmanned intelligent electronic devices are gradually being applied to various fields, such as intelligent assisted-driving cars, 5G smart base stations, multi-field remote-controlled drones, and unmanned transportation systems in coal mines. However, electromagnetic waves emitted by these devices interfere with each other to form electromagnetic pollution, affecting equipment stability and human health, while posing potential dangers to the human body. Developing high-performance materials resistant to electromagnetic interference is crucial for the stable operation of intelligent electronic devices [1,2]. *MAMs* possess advantages such as high absorption capacity, broadband performance, low thickness, and strong stability. They dissipate electromagnetic wave energy through specific mechanisms, thereby absorbing the electromagnetic wave, effectively addressing the issue of electromagnetic pollution [3,4,5]. These materials play a pivotal role in the field of national defense and security [6,7,8].

The two key factors affecting the performance of *MAM*s are impedance matching and attenuation characteristics. When an electromagnetic wave impinges upon the material’s surface, impedance matching determines the amount of penetration into the material’s interior. The closer the impedance matching value to 1, the greater the electromagnetic wave penetration. Attenuation characteristics, or the material’s loss capacity, categorize *MAMs* based on their loss mechanisms: resistive loss materials, dielectric loss materials, and magnetic loss materials [9,10]. In the context of electrical current passage, resistive loss materials undergo a significant number of collisions between free electrons within the material, resulting in the conversion of electrical energy into thermal energy. This phenomenon predominantly occurs in materials with high electrical conductivity, such as graphene, carbon nanotubes, and conductive polymers. On the other hand, dielectric loss materials, which contain few free electrons, undergo molecular friction, ionization, relaxation, and other processes when exposed to electromagnetic microwaves, without generating a macroscopic current, leading to a certain loss of energy. Examples include materials like Al_2_O_3_ and SiO_2_. Magnetic loss primarily encompasses mechanisms such as hysteresis loss, eddy current loss, natural resonance, and domain wall resonance. Materials subjected to magnetic loss undergo processes of magnetization or demagnetization in alternating electromagnetic fields, with a portion of the energy being converted into thermal energy [11,12]. Examples include ferrites and nickel-cobalt alloys. As the frequency of the alternating electromagnetic field increases, magnetic loss generally also increases, primarily due to natural resonance and domain wall resonance. Based on this mechanism, the selection of lossy materials is pivotal to the realization of high-performance *MAMs*. Typically, composites are prepared using two or three lossy materials, amplifying the loss synergism, and enhancing the radar-absorbing effect. Ferrites possess numerous advantages, including excellent magnetic permeability and magnetic loss, outstanding temperature and chemical stability, low cost, and strong microwave-absorbing performance.

Ferrites can be broadly classified into three types, based on their crystal structure: spinel (cubic system), garnet (cubic system), and magnetorheological (hexagonal system). The spinel type of ferrite has the chemical formula MeFe_2_O_4_, where Me represents divalent metal ions such as Co^2+^, Cu^2+^, and Ni^2+^, with oxygen ions arranged in a face-centered cubic (fcc) dense packing. Spinels exhibit high magnetic saturation induction, making them one of the most studied and widely applied types. The garnet structure has the formula R_3_Fe_5_O_12_, where R represents trivalent rare earth ions such as Y^3+^ and Sc^3+^. The performance of garnets is influenced by their crystal structure, and the properties of this material can be adjusted by varying the type of R. The magnetorheological type has the formula MeFe_12_O_19_, where Me is typically Ba^2+^, with substitutions for Mn, Zn, Al, etc. This type of ferrite exhibits high magnetic anisotropy and a natural resonant frequency, making it an effective *MAM* in the centimeter wave band.

The research on ferrite *MAMs* can be traced back to the 1940s. Due to issues such as poor impedance matching, a single mechanism for magnetic loss, narrow absorption bandwidth, and high density, the materials are greatly limited in their applications. Modifications to ferrites are often achieved by doping metal ions, which cause distortions and defects in the internal structure. These defects can act as polarization centers, leading to electron shifts and enhanced loss to electromagnetic waves. The superior performance is attributed to the intricate design of the microstructure. By regulating and optimizing the material’s microstructure, we can enhance and improve various properties of the material, including microwave absorption, physical, chemical, and mechanical properties. This results in the preparation of *MAMs* with high strength, wide bandwidth, low thickness, and good stability. In recent years, numerous studies have been conducted by scholars on ferrite-based *MAMs*. This article, from the perspectives of the microstructure, preparation methods, and composition, summarizes the research progress and challenges regarding the microstructure of ferrite-based *MAMs*, and points out the future development trends.

## 2. Sheet Structure

The sheet structure possesses a substantial surface area, with each node interconnected to form a unified network entity, providing additional anchor points. Even if some node atoms undergo substitution, it barely affects the overall structure, yet still achieves the goal of modification. Graphene is a prototypical material of the sheet structure, exhibiting a planar hexagonal honeycomb structure, with a pronounced dielectric loss and excellent conductivity. Boundary groups and planar defects can enhance the conductivity loss. The sheet structure is suitable as a carrier for nanoparticles, enabling the preparation of various functional composite materials. For instance, on graphene oxide sheets supported by polymers, the synthesized nanorods are enhanced with carbon nanotubes and chitosan, representing a promising bone filling material. Moreover, it has been extensively studied and applied in fields such as electromagnetic microwave absorption [13,14], biomedicine [15,16], biosensors, and supercapacitors [17,18]. Assembling graphene with ferrite nanoparticles can effectively achieve a complementation of dielectric loss and magnetic loss, enhancing the electromagnetic wave loss capability.

**Figure 1:**
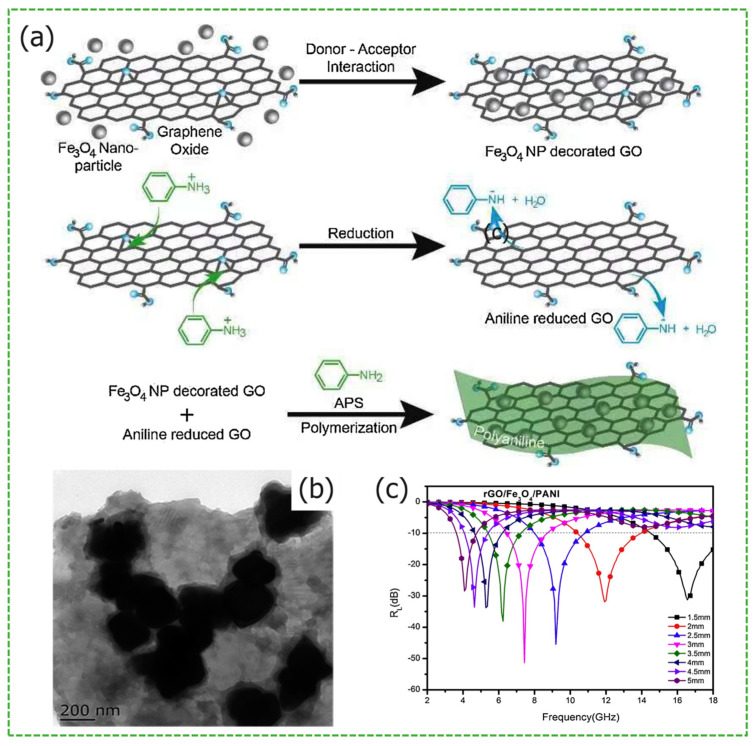

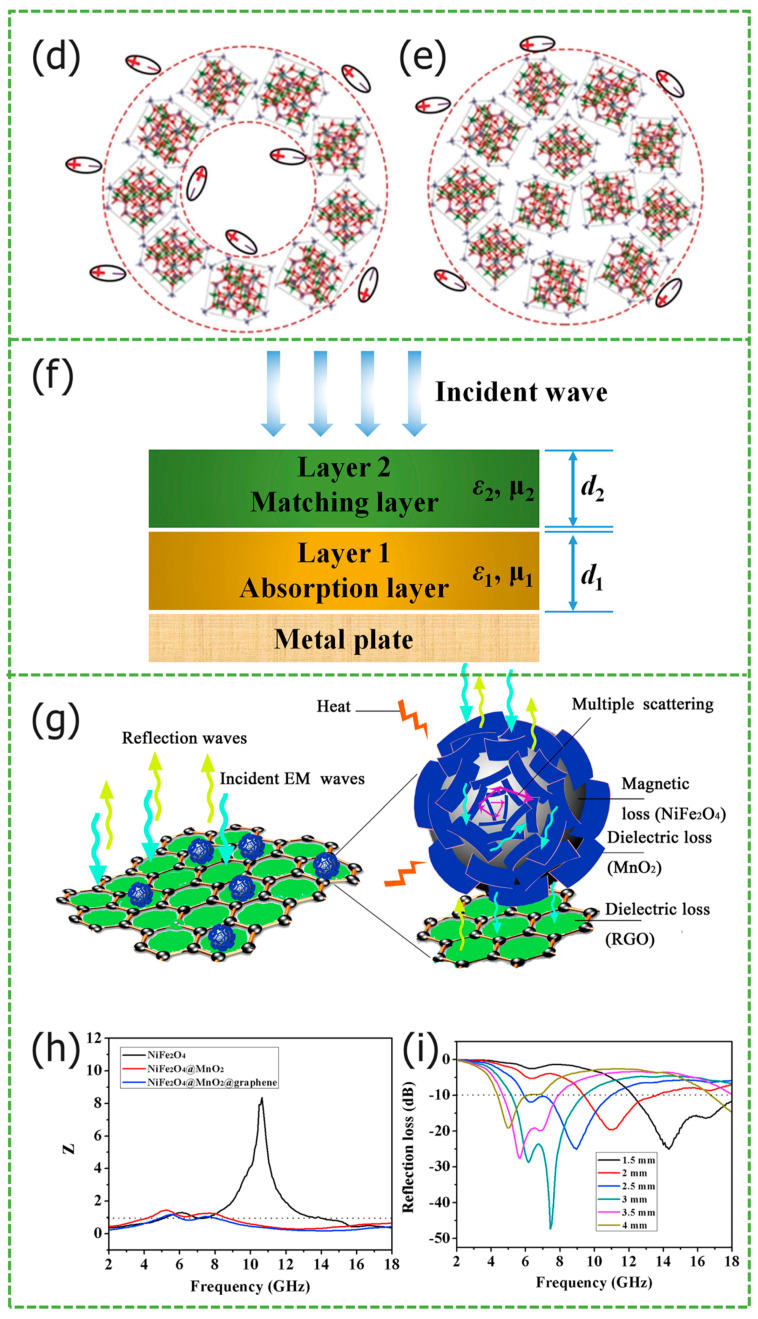
Graphical summary of sheet structure *MAMs*. (**a**) The synthesis schematic diagram, (**b**) TEM image, and (**c**) the *RL* for RGO/Fe_3_O_4_/PANI. Reproduced with permission [13] Copyright 2020, Elsevier B.V. (**d**,**e**) The dielectric polarizations in hollow CoFe_2_O_4_ NPs and solid CoFe_2_O_4_ NPs, respectively. Reproduced with permission [25]. Copyright 2018, Royal Society of Chemistry. (**f**) Schematic diagram of double-layer MAM. Reproduced with permission [29] Copyright 2017, Elsevier B.V. (**g**) Schematic illustration for absorption mechanism, (**h**) impedance matching, and (**i**) the RL curves of NiFe_2_O_4_@MnO_2_@graphene. Reproduced with permission [27] Copyright 2016, Elsevier B.V.

Graphene oxide (GO) possesses a surface rich in oxygen-containing functional groups, exhibiting high chemical reactivity. Sun et al. utilized a hydrothermal method to synthesize a ternary composite material, copper-cobalt-nickel ferrite@GO@polyaniline (PANI) [19], successfully prepared a coating fabric, using aqueous polyurethane as the matrix. When the ternary composite material is applied in an amount of approximately 40%, the fabric thickness is 2.0 mm and the *RL_max_* at 10.8 GHz is −33 dB, with an effective absorption bandwidth (*EAB*: RL < −10 dB) of approximately 6.95 GHz. The shielding performance can reach −47 dB within the frequency range of 300 kHz to 3.0 GHz. After chemical oxidation and stripping of graphite powder, reduced graphene oxide (RGO) sheets are obtained, which exhibit properties similar to those of graphene. However, RGO typically contains more defects and other impurities, leading to a higher conductive loss capacity, making it more suitable as a doping substrate compared to graphene. Wang and colleagues synthesized RGO@Fe_3_O_4_@PANI nanocomposite material [13] by reducing GO with aniline. From Figure 1a the synthesis schematic diagram and (b) TEM image of RGO/Fe_3_O_4_/PANI, the anchoring of ferrite particles and PANI onto the surface of GO sheets leads to magnetic losses and enhanced dielectric losses. Molecular dynamics simulations indicate a strong interaction between carboxyl groups at the edges of graphene and iron atoms in the ferrite. When the graphene sheet is introduced from a parallel direction onto the Fe_3_O_4_(111) surface, the interfacial interaction energy is low, making it easier to form a smooth single-layer structure. In an alternating electromagnetic field, electrons are displaced, resulting in interfacial polarization. Figure 1c shows the *RL_max_* of RGO@Fe_3_O_4_@PANI at 7.4 GHz is −51.5 dB, with an *EAB* of 4.2 GHz. Compared to graphene-based composite materials, its microwave absorption performance is significantly improved.

Doping can alter the lattice structure of ferrites, modulating their electromagnetic parameters and properties such as magnetic anisotropy [8,9]. Transition metal ions like Ni, Co, and Zn, as well as rare earth elements like Ce, La, and Nd, when doped, cause changes in the lattice structure of ferrites, leading to lattice distortion, disruption of exchange interactions, and local chemical disorder. This increases internal defects, adjusting the electromagnetic parameters and properties such as magnetic anisotropy, and enhances the microwave absorption capability of the ferrites [3,7]. The unpaired 4f electrons and strong spin-orbit coupling of the rare earth element Ce ions enhance the dielectric properties of ferrites, while increasing magnetic anisotropy improves the coercivity of the materials. Under electromagnetic fields, induced dipole polarization enhances the absorption intensity of electromagnetic waves [20]. The incorporation of non-magnetic transition metal ions, such as Zn^2+^, can reduce the coupling between magnetic ions [21], decrease the coercivity, and increase the saturation magnetization, leading to a favorable attenuation effect for high-frequency and ultra-high-frequency signals. Chireh et al. substituted Fe^3+^ in LiFe_5_O_8_ with Sr^2+^ and Co^2+^. Due to the electronic transition between Fe^3+^ and Fe^2+^, magneto-crystalline anisotropy, exchange anisotropy, and shape anisotropy were caused by substitution of Sr^2+^ and Co^2+^, resulting in higher and lower saturation magnetization and coercivity fields for RGO/LiSr_0.25_Fe_4.75_O_8_ and RGO/LiCo_0.25_Fe_4.75_O_8_ nanoparticles than those of pure LiFe_5_O_8_ ferrite. The magnetic parameter test results show that partial substitution resulted in a larger complex dielectric constant, and the RGO/LiSr_0.25_Fe_4.75_O_8_ and RGO/LiCo_0.25_Fe_4.75_O_8_ nanocomposite materials [22] exhibit a broader *EAB*, with varying degrees of improved *RL_max_* compared to LiSr_0.25_Fe_4.75_O_8_ and LiCo_0.25_Fe_4.75_O_8_. The RGO/LiCo_0.25_Fe_4.75_O_8_ composite material, with a sample thickness of 3 mm, exhibits a *RL_max_* of −46.80 dB at 13.20 GHz, and an *EAB* of 6.80 GHz (10.52–17.32 GHz). In contrast to the heat treatment and polymerization methods of Chireh et al., Shu et al. utilized a simpler solvothermal method to synthesize the RGO/ZnFe_2_O_4_ hybrid nanocomposite material [23], with a *RL_max_* of −41.1 dB when the sample thickness is 2.5 mm. The superior microwave absorption performance of RGO//LiCo_0.25_Fe_4.75_O_8_ and RGO/ZnFe_2_O_4_ indicates that it is feasible to anchor sheet-like RGO to ferrite nanoparticles.

Li et al. substituted Fe^3+^ with Nd^3+^, utilizing solid solution and hydrothermal synthesis to produce the RGO/Ni_0.4_Co_0.2_Zn_0.4_Nd_x_Fe_2−x_O_4_ composite materials [24]. As x gradually increases, the *RL_max_* deepens, and at x = 0.06, the RGO/Ni_0.4_Co_0.2_Zn_0.4_Nd_0.06_Fe_1.94_O_4_ composite material exhibits a *RL_max_* of −58.33 dB at 12.2 GHz, with a matching thickness of 2.33 mm, an *EAB* of 7.5 GHz (5.0–12.5 GHz), and a further enhanced microwave absorption performance.

Zhang et al. synthesized a composite material of RGO/CoFe_2_O_4_/SnS_2_ using the hydrothermal method [25]. Figure 1d,e shows the dielectric polarizations in hollow CoFe_2_O_4_ NPs and solid CoFe_2_O_4_ NPs. The material exhibited a saturation magnetization (MS) of 22.9 emu/g and a remanence (Mr) of 1.9 emu/g, preserving the excellent magnetic properties of CoFe_2_O_4_. The *RL_max_* of the sample at 16.5 GHz reached −54.4 dB, with an *EAB* spanning the entire X-band, up to 12.0 GHz (6.0–18.0 GHz). In Wang et al.’s work, the synthesized NiFe_2_O_4_@MnO_2_@graphene composite material [26] exhibited good impedance matching, primarily due to the increased contact area with air, caused by the gap between MnO_2_ and graphene, enhancing impedance matching. The MS of NiFe_2_O_4_ was 54.8 emu/g, and the *RL_max_* of the composite sample at 7.4 GHz reached −47.4 dB. It is evident that ferrites such as CoFe_2_O_4_ and NiFe_2_O_4_ doped with Ni and Co, improve the electromagnetic microwave absorption capability of the composite material. Yan et al. prepared RGO-PANI-NiFe_2_O_4_, RGO-polypyrrole (PPy)-NiFe_2_O_4_, and RGO-3,4-PEDOT-NiFe_2_O_4_ composite materials [27]. From Figure 1g–i, the NiFe_2_O_4_ particles impart superparamagnetic to the composite materials, achieving peak *RL_max_* of −49.7 dB, −44.8 dB, and −45.4 dB, respectively. Gao et al. synthesized a BiFeO_3_/RGO composite material through a hydrothermal reaction [28], achieving a *RL_max_* of −46.7 dB, an *EAB* of 4.7 GHz (12.0–16.7 GHz), and a matching thickness of 1.8 mm.

Unlike others, Min et al. synthesized BaFe_12_O_19_/graphite composites using BaFe_12_O_19_ and graphite nanosheets as matching and absorbing layers, respectively [14]. However, the improvement in microwave absorption performance by the materials was very limited, with *RL_max_* of only −26 dB and narrow *EAB* at a sample thickness of 2.5 mm. The main reason for this result is that the incidence and absorption of electromagnetic microwaves are almost synchronized, and the poor impedance matching of the BaFe_12_O_19_ layer results in most of the electromagnetic microwaves being reflected, with only a small amount of them incident on the graphite layer being absorbed. A similar design was used in the work of Liu et al. Co_0.2_Ni_0.4_Zn_0.4_Fe_2_O_4_ (CNZF) ferrite and RGO were used as matching and absorbing layers for *MAM* in Figure 1f, respectively [29]. The CNZF exhibits good impedance matching, the double-layer *MAM* has a *RL_max_* of −49.5 dB at 16.9 GHz, and an *EAB* of 6.0 GHz at a mass fraction of 30%, with thickness of 2.5 mm, which is a significant enhancement in the absorbing performance.

The moderate increase in defects and functional groups in the lamellar structure generates more electromagnetic microwave loss mechanisms, enhances multiple synergistic losses, and improves the microwave-absorbing performance.

## 3. Layered Structure

The layered structure can increase the contact area between materials. When the dielectric constants and conductivities of two materials differ, charge accumulation occurs at the contact interface. The accumulation of positive and negative charges intensifies electron shifts, enhancing the interfacial polarization effect. MXene is a prototypical material with a layered structure, composed of alternating carbon layers and transition metal layers, primarily connected by van der Waals forces between layers. The flexible selection of M and X elements not only endows MXene with superior conductivity and dielectric loss characteristics, but also provides a broader range of tunability. In addition to MXene, stacked graphite and graphene can also form layered structures, and anchoring ferrite nanoparticles between layers is a common approach. This results in a complementation of dielectric loss and magnetic loss.

**Figure 2:**
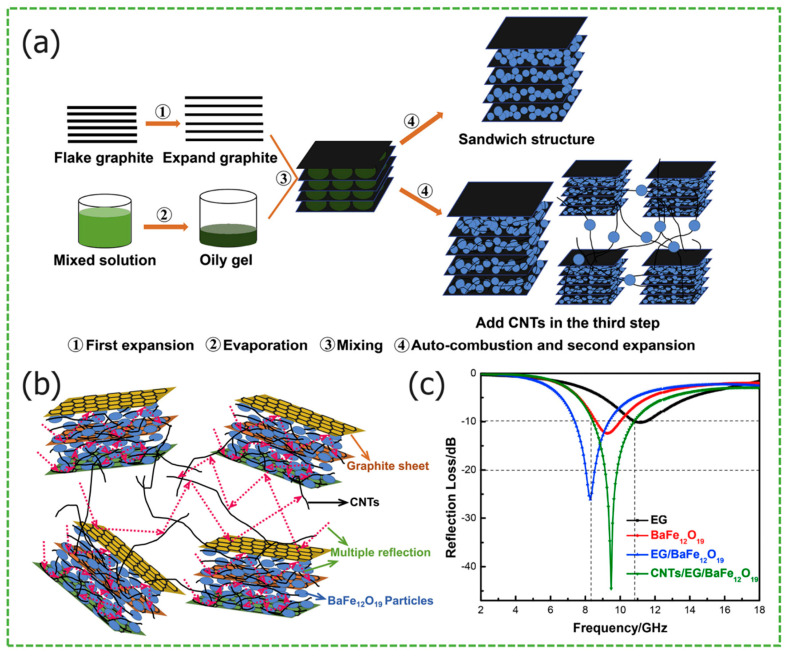

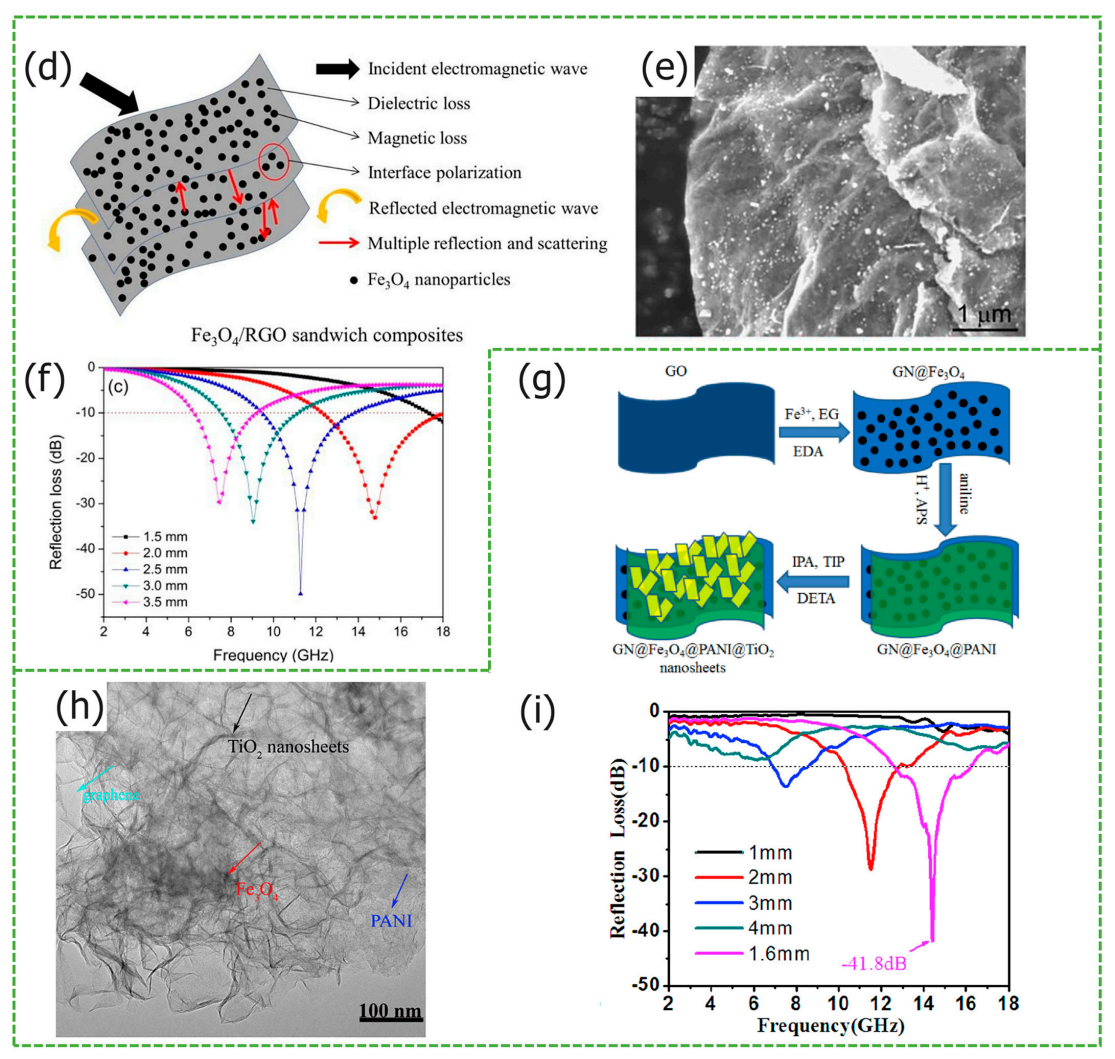
Graphical summary of layered structure *MAMs*. (**a**) Synthesis schematic diagram, (**b**) absorption mechanism, and (**c**) the *RL* curves of sandwich CNT/EG/BF nanocomposite. Reproduced with permission [30] Copyright 2017, Elsevier B.V. (**d**) Schematic diagram of absorption mechanism, (**e**) SEM image, and (**f**) the *RL* curves of Fe_3_O_4_/RGO-3 sandwich composites. Reproduced with permission [31] Copyright 2023, Elsevier Inc. (**g**) Schematic illustration of the fabrication, (**h**) TEM image, and (**i**) the *RL* curves of the GN@Fe_3_O_4_@PANI@TiO_2_ nanosheets. Reproduced with permission [32] Copyright 2016, Elsevier B.V.

Zhao et al. synthesized carbon nanotubes/expanded graphite/BaFe_12_O_19_ (CNT/EG/BF) composite material, using an in-situ sol–gel self-combustion method. From Figure 2a synthesis schematic diagram can be seen that carbon nanotubes serve as a conductive network, connecting the expanded graphite layers and the interlayer bonds of the expanded graphite with BaFe_12_O_19_ [30]. According to the absorption mechanism of sandwich CNT/EG/BF in Figure 2b, Figure 2c shows the *RL_max_* of −45.8 dB of the sample, with an *EAB* of 4.2 GHz, and a matching thickness of only 1 mm. Compared with the functionally layered BaFe_12_O_19_/graphite composites, the microwave-absorbing properties are dramatically improved, taking advantage of the combination of expanded graphite and BaFe_12_O_19_. In the work of Li et al., the synthesized Fe_3_O_4_/RGO composites with a similar sandwich structure have obvious advantages [31]. From Figure 2d schematic diagram of absorption mechanism and (e) SEM image of Fe_3_O_4_/RGO-3 sandwich composites, the layered structure not only effectively inhibits the aggregation of ferrite particles, but also induces the particles to be uniformly distributed on the surface of RGO, producing interfacial polarization. Figure 2f shows the *RL_max_* of −49.9 dB of samples, and *EAB* covers 5.7 GHz. Liu et al. introduced TiO_2_ and PANI materials to graphene, and synthesized graphene@Fe_3_O_4_@PANI composites [32], it decorated with random vertically distributed TiO_2_ nanosheets. From Figure 2g schematic illustration of the fabrication and (h) TEM image of composites, TiO_2_ further promotes interfacial polarization and impedance matching. Figure 2i shows that when the paraffin doping was 50 wt%, the composites exhibited a *RL_max_* of −41.8 dB at 14.4 GHz, with an *EAB* of 3.5 GHz and a matching thickness of only 1.6 mm. Lei et al. prepared two-dimensional Ti_3_C_2_T_x_ using HF etching, which was combined with ferrite particles, synthesizing Ti_3_C_2_T_x_/Co-doped NiZn ferrite (CNZFO)/PANI composites [33]. The ferrite particles and PANI chains were attached to the Ti_3_C_2_T_x_ structure, contributing to the synergistic enhancement of the loss mechanism. Compared with CNZFO and Ti_3_C_2_T_x_, the Ti_3_C_2_T_x_/CNZFO/PANI composite exhibits a deeper *RL_max_* of −37.1 dB, a wider *EAB* of 4.1 GHz (8.2–12.3 GHz) at 10.2 GHz, and a matched thickness of 2.2 mm.

Li et al. and Guo et al. used similar methods to synthesize Ti_3_C_2_T_x_/Ni_0.5_Zn_0.5_Fe_2_O_4_ [34] and Ti_3_C_2_T_x_/Ni_0.6_Zn_0.4_Fe_2_O_4_(NZFO) composites [35], respectively. The former Ti_3_C_2_T_x_ with 5 wt % doping showed a *RL_max_* of −42.5 dB at 13.5 GHz, while the latter Ti_3_C_2_T_x_/NZFO-2 showed a *RL_max_* of −66.2 dB at 15.2 GHz, with an *EAB* of 4.74 GHz, and a thickness of only 1.609 mm. The obvious difference in the *RL_max_* of the two composites may be due to the following factors: the significant ferrite lattice changes due to the different doping amounts of Ni and Zn, as well as the different composite methods used. Although MXene suffers from the problem of self-stacking, the interlayer is prone to agglomeration and re-stacking. By introducing ferrite particles, the above problems can be effectively solved by weakening the excessive conductivity and increasing the magnetic loss capability.

In the study of Swapnalin et al., it was found that MXene anchored moderate CoFe_2_O_4_ ferrite particles, increasing the dielectric constant and permeability of Ti_3_C_2_T_x_@CoFe_2_O_4_ composites [36], probably due to the formation of many defective dipoles by the incorporation of CoFe_2_O_4_, which triggers an inhomogeneous local charge distribution. Polyvinyl Butyral/Ba_3_Co_2_Fe_24_O_41_/Ti_3_C_2_ MXene composites were synthesized by Yang et al. [37]. MXene nanosheets significantly reduce the saturation magnetization, and varying filler content can optimize electromagnetic parameters, thereby improving the microwave absorption properties. The *RL_max_* of composites is −46.3 dB at 5.8 GHz.

The layered structure has a high surface-area-to-volume ratio, and the gaps between the layers promote the adsorption of ferrite nanoparticles, enhancing the absorption performance of composite materials.

## 4. Core–Shell Structure

The core-shell structure is typically achieved through various techniques [38,39], such as solvothermal, templated, hydrothermal, or modified Stöber methods, by the orderly assembly of one or more materials. The interplay of atomic forces promotes the tight encapsulation of the core by the outer layer material, resulting in a layered core–shell structure in which all or part of the core’s surface is enveloped. The properties of each core and shell, as well as the interface region formed by their interactions, collectively determine the nature and performance of the core–shell structure. For instance, by establishing a unique core–shell heterojunction structure, S@NiFe-LDH enhances the photocatalytic activity and stability of the catalyst [40]. Core–shell materials have been extensively studied and applied in various fields such as electromagnetic microwave absorption, batteries [41,42,43], supercapacitors [44,45,46], sensors [47], biomedicine [48], semiconductors [49,50], and stain and corrosion prevention [51]. Ferrite microspheres are wrapped on the surface of the shell, and electromagnetic waves are incident into the core–shell structure; multiple reflections and scatterings occur within it, resulting in tight encapsulation between materials and enhanced electromagnetic synergies, leading to a loss in electromagnetic wave energy. Based on their microscopic morphology and internal composition, core–shell structures are classified into four types: solid core–shell structures, hollow core–shell structures, yolk–shell structures, and non-spherical core–shell structures.

### 4.1. Solid Core-Shell Structure

The solid core-shell is the most fundamental type of core-shell structural system, where the outer layer material directly wraps around the core, forming a more polarized interface structure. The shell material usually has higher mechanical strength than the core material, avoiding oxidation or damage to the core material.

**Figure 3:**
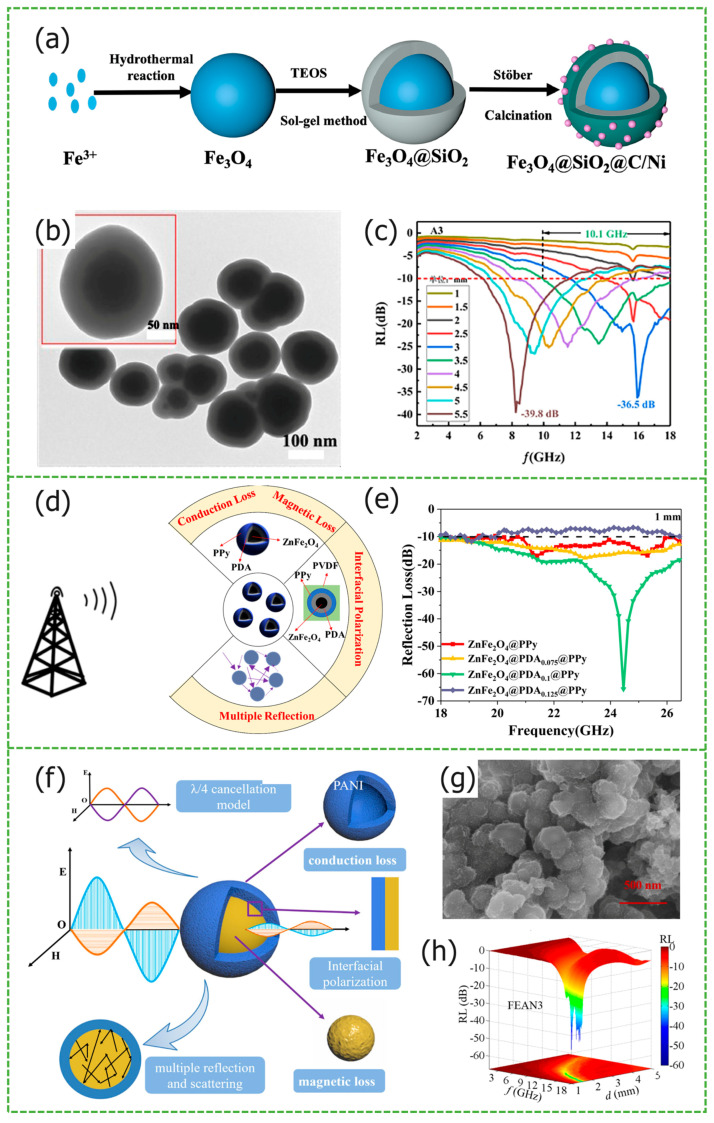
Graphical summary of solid core–shell structure *MAMs*. (**a**) Schematic illustration of the preparation, (**b**) SEM image, and (**c**) the *RL* value of Fe_3_O_4_@SiO_2_@C/Ni. Reproduced with permission [53] Copyright 2023, Elsevier B.V. (**d**) Absorption mechanism and (**e**) *RL* maps of ZnFe_2_O_4_@PDA_0.1_@PPy. Reproduced with permission [58] Copyright 2021, Springer US. (**f**) Schematic diagram of absorption mechanism, (**g**) SEM image, and (**h**) 3D *RL* contour maps of FEAN3. Reproduced with permission [54] Copyright 2022, Elsevier B.V.

Shi et al. utilized dopamine as a carbon source, synthesizing Fe_3_O_4_@C composite materials through continuous high-temperature carbonization [38]. The microspheres exhibit a layered structure, with the carbon shell encapsulating Fe_3_O_4_ microspheres, forming a multi-interface heterostructure, and resulting in a synergistic electromagnetic interaction. This approach effectively prevents aggregation among magnetic core microspheres, enhancing magnetic responsiveness. In contrast to the Fe_3_O_4_@C microspheres prepared by Du et al., using in situ polymerization and high-temperature carbonization [52], which have a *RL_max_* of approximately −36 dB and a narrow *EAB*, the layered Fe_3_O_4_@C microspheres, with a thickness of 2.0 mm, achieve a *RL_max_* of −55.4 dB and an *EAB* spanning 9.5 GHz (8.5–18 GHz), significantly enhancing their microwave absorption performance. Using ferrite microspheres as the matrix, selecting different materials as carbon layers is a common method for preparing solid core–shell materials. Based on Fe_3_O_4_@C, Jia et al. introduced Ni atoms and SiO_2_, which play the roles of enhancing the magnetic loss capability and optimizing the impedance matching, respectively. The preparation diagram is shown in Figure 3a [53], Figure 3b SEM image shows Fe_3_O_4_@SiO_2_@C/Ni composites with a double-core-shell structure, where the electromagnetic wave multiple reflection and scattering loss is further enhanced. The Fe_3_O_4_ integrity is well preserved due to the protective effect of SiO_2_. Figure 3c electromagnetic parameter test displays the *RL_max_* of −38.9 dB and *EAB* reaches 10.1 GHz for Fe_3_O_4_@SiO_2_@C/Ni, at a thickness of 3.5 mm. Due to the alternating benzene rings and nitrogen atoms on the carbon chain of conductive polymer PANI, it has special electrical and photoelectric properties, and is widely used in the fields of batteries and capacitors. Wang et al. synthesized Fe_3_O_4_@PANI core-shell nanorods [54]. In Figure 3f, the dielectric loss of conductive PANI and the magnetic loss of Fe_3_O_4_ nanorods effectively complement each other. However, From Figure 3g SEM image can be seen that particles stick together. The Fe_3_O_4_@PANI show that the *RL_max_* at 17.3 GHz is −55.5 dB, and the matching thickness is only 1.6 mm from Figure 3h. In order to pursue *MAMs* with higher strength, wider bandwidth, etc., they are usually constructed using Fe_3_O_4_@C. On this basis, other dielectric materials and magnetic loss materials are introduced to further enhance the polarization between interfaces. Zha et al. used nitrogen doping and Ti_3_C_2_T_x_ composite to prepare Fe_3_O_4_/NC@MXene (FNCM) composite materials [55]. The interface polarization between Ti_3_C_2_T_x_ and ferrite microspheres increases, and nitrogen doping causes the charge distribution in the carbon layer to rearrange, enhancing dipole polarization and conductivity loss. The *EAB* of the sample FNCM-2 is 7.32 GHz and the *RL_max_* is −54.41 dB at a thickness of 2 mm. TiO_2_ has a high dielectric constant. Shi et al. introduced black TiO_2−x_ into Fe_3_O_4_@TiO_2_ to prepare Fe_3_O_4_@b-TiO_2−x_ [56]. Compared to the traditional Fe_3_O_4_ and Fe_3_O_4_@TiO_2_ microspheres, this novel core–shell heterostructure significantly enhances the microwave-absorbing properties. With a matching thickness of 2.9 mm, Fe_3_O_4_@b-TiO_2−x_ achieves a *RL_max_* of −47.6 dB, and the *EAB* reaches up to 13.0 GHz.

Chen et al. prepared C@Ni_x_Co_1−x_Fe_2_O_4_ composite nanospheres including NiFe_2_O_4_, cobalt-doped nickel ferrite, nickel-cobalt ferrite, nickel-doped cobalt ferrite, and various types of nickel-doped cobalt ferrite using a solvothermal reaction [57]. As the Co content increases, the crystal structure parameters change, and lattice distortion and local chemical disorder lead to a gradual increase in the coercivity of the composite nanospheres. C@CoFe_2_O_4_ exhibits the highest magnetization, reaching 332.1 Oe. The electromagnetic parameter test results showed that, with a Ni doping ratio of 0.75 and Co doping ratio of 0.25, the prepared C@Ni_0.75_Co_0.25_Fe_2_O_4_ nanospheres have the strongest microwave-absorbing performance: the *RL_max_* was −51 dB, the *EAB* was 3.3 GHz, and the corresponding matching thickness was only 1.9 mm. Ge et al. prepared ZnFe_2_O_4_@polydopamine(PDA)@PPy composites using the hydrothermal method and in situ polymerization of PDA [58], Figure 3d shows the synergistic effect of multiple loss mechanisms, when dopamine hydrochloride was used in the amount of 0.1 g, the *EAB* covered the range of 18–40 GHz, and the *RL_max_* at 24.46 GHz was −65.66 dB from Figure 3e.

The tight combination of core and outer core materials results in a large amount of interface polarization in the core-shell structure material, where electrons gather and enhance the loss in electromagnetic waves.

### 4.2. Hollow Core-Shell Structure

In a hollow core-shell structure, the outer layer material wraps around the core, forming a hollow area in the middle, effectively reducing the mass of the core-shell structure. Increasing the contact area between the hollow area and the air, and optimizing impedance matching, are beneficial for the occurrence of multiple reflections and scattering of incident electromagnetic waves.

**Figure 4:**
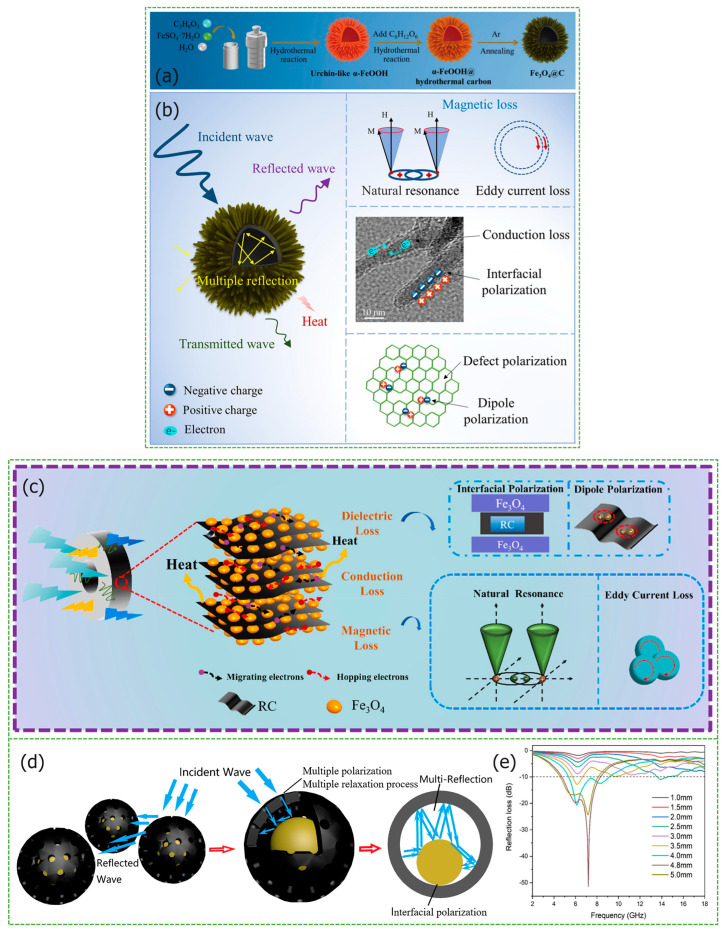
Graphical summary of hollow core-shell structure *MAMs*. (**a**,**b**) Schematic diagram of the preparation process and absorption mechanisms of Fe_3_O_4_@C. Reproduced with permission [66]. Copyright 2023, Elsevier Inc. (**c**) The potential absorbing mechanisms of Fe_3_O_4_ NPs@RC. Reproduced with permission [63]. Copyright 2023, Elsevier Ltd. and Techna S.r.l. (**d**,**e**) Absorption mechanism and *RL* curves of ZnFe_2_O_4_@porous hollow carbon microspheres. Reproduced with permission [60]. Copyright 2021, Elsevier Inc.

Similar to the preparation method of solid core-shell structures, the synthesis of hollow core–shell structures are usually carried out in the Fe_3_O_4_@C. On this basis, other dielectric materials and magnetic loss materials are introduced. However, there are slight differences in the use of raw materials and synthesis methods, resulting in cavity structures. In the work of Zhu et al., Fe_3_O_4_@porous carbon composites with hollow core-shell structures were prepared [59]. The porous structure optimizes impedance matching, enhancing the specific surface area and facilitating the dissipation of incident electromagnetic wave energy. The carbon-derived sample FC-700, synthesized at 700 °C, exhibits outstanding microwave absorption properties, achieving a *RL_max_* of −50.05 dB at 1.8 mm thickness and an *EAB* of 5.20 GHz. Mainly through carbonization, amorphous carbon is generated and there are many defects. Chai et al. obtained hollow microspheres by etching silica with hydrofluoric acid, and synthesized ZnFe_2_O_4_@C composite materials through self-assembly and in situ preparation techniques. Additionally, Figure 4d shows that numerous uniform micropores are formed on the surface, improving impedance matching [60]. The carbon microspheres exhibit a porous hollow structure, leading to multiple reflections and scatterings of electromagnetic waves within the microspheres, coupled with the formation of numerous uniform micropores on the surface, which improves impedance matching. Notably, for the sample ZFO@C-1, with a thickness of 4.8 mm, the *RL_max_* at 7.2 GHz is −51.43 dB, and the *EAB* is 3.52 GHz from Figure 4e. The residual carbon from the fine slag of coal gasification, characterized by a distinct graphitized structure [61,62], was utilized by Gao et al. as a cost-effective carbon source to synthesize Fe_3_O_4_@residual carbon composites [63]. The absorption mechanism is shown in Figure 4c. When the filler content is 40 wt%, the thickness of sample ranges from 1.5 mm to 5 mm, and the *EAB* covers Ku, X, and C bands, with a *RL_max_* of −32.6 dB at a thickness of 2.0 mm. Dong et al. synthesized a composite material consisting of Fe_3_O_4_@PPy@RGO [64]. Flake RGO connects hollow microspheres, synergistically optimizing dielectric and electromagnetic losses, and enhancing absorption performance. The *RL_max_* of the sample with 1.89 mm thickness is −61.20 dB.

The biomimetic sea urchin-shaped hollow core-shell structure is lightweight, and the gaps between the fine needles increase the specific surface area, optimizing impedance matching. The sea urchin-shaped SrFe_12_O_19_ prepared by Chen et al. [65] has a *RL_max_* of −22.8 dB and an *EAB* of 5.6 GHz (12.4–18.0 GHz) at 15.1 GHz, with a thickness of 3 mm. Wu et al. chose to use α-FeOOH as a precursor to synthesize sea urchin-like structures, using hydrothermal and annealing methods for a Fe_3_O_4_@C composite material, the preparation process is shown in Figure 4a [66]. When the mass ratio of α-FeOOH to glucose is 1:1, the Fe_3_O_4_@C with a thickness of 3.23 mm shows an *RL_max_* of −73.5 dB. The sea urchin-like core-shell structure enhances interfacial polarization, leading to an electron shift in an alternating electromagnetic field. The absorption mechanism is displayed in Figure 4b, which includes multiple loss mechanisms.

Zhang et al. prepared CoFe_2_O_4_@carbon nanotube composite materials by replacing Fe^3+^with Co^2+^ through the chemical vapor precipitation method. The carbon nanotubes are coated on the surface of CoFe_2_O_4_ hollow microspheres [67]. The *RL_max_* of the sample at 11.7 GHz is −32.8 dB, with a thickness of merely 2 mm.

An appropriate number of voids can reduce the quality of *MAMs*, optimize impedance matching, and promote the strongest performance of composite materials.

### 4.3. Yolk-Eggshell Structure

Yolk–eggshell is a structure that lies between a solid core-shell and a hollow core-shell, similar to an egg. There is a certain gap between the inner and outer cores, while maintaining a solid structure inside. Under external forces, the internal solid has a certain degree of mobility, offsetting external work done.

**Figure 5:**
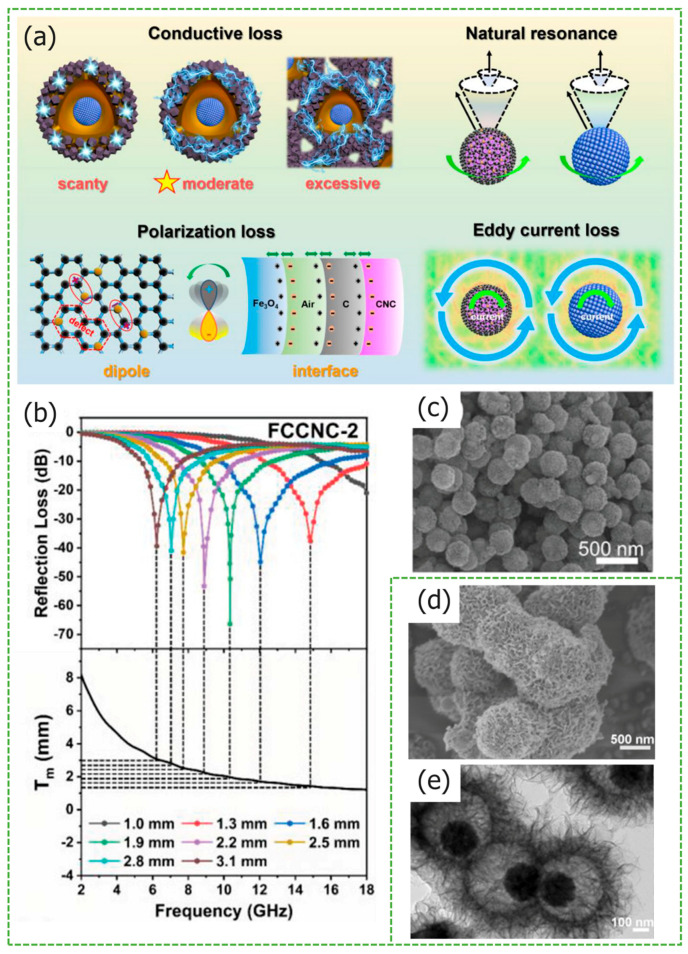
Graphical summary of yolk-eggshell structure *MAMs*. (**a**) The specific electromagnetic mechanism of absorption and (**b**) the *RL* curves of FCCNC-2. (**c**) FESEM images of yolk-shell Fe_3_O_4_@C@Co/N-doped C. Reproduced with permission [71] Copyright 2023, Wiley. (**d**) FESEM and (**e**) TEM images of the Fe_3_O_4_@TiO_2_ yolk–shell microspheres. Reproduced with permission [69] Copyright 2013, Easton, Pa. [etc.] American Chemical Society [etc.].

Liu et al. synthesized Fe_3_O_4_@SiO_2_ core–shell microspheres, using the enhanced Stöber method. Building upon prior research, they found that a silica coating on Fe_3_O_4_ particles could effectively modify their surface properties [68]. Subsequently, they hydrothermally deposited SnO_2_, resulting in a Fe_3_O_4_@SnO_2_ double-shell structure with a yolk-like structure [39]. These microspheres exhibit a high specific surface area and uniform dimensions, which is attributed to the favorable electromagnetic interaction between the core and shell. When the sample MTO-3 is 2 mm thick, its *RL_max_* at 7 GHz is 36.5 dB, with an *EAB* spanning from 2 to 18 GHz. The reflection loss is consistently below −20 dB. Compared to Fe_3_O_4_ particles, it demonstrates superior microwave absorption performance. In another study by Liu et al., by replacing SnO_2_ with TiO_2_, Fe_3_O_4_@TiO_2_ layered yolk–shell microspheres were prepared using a template method, including in various sizes [69]. Figure 5d,e shows the presence of pores between the outer TiO_2_ nanosheets, resulting a large specific surface area, optimizing impedance matching and allowing more electromagnetic waves to be incident on the inside of the yolk shell. At a thickness of 2 mm at 7 GHz, Fe_3_O_4_@TiO_2_ exhibited an *EAB* of nearly 14.5 GHz, significantly surpassing Fe_3_O_4_ and Fe_3_O_4_@SiO_2_@TiO_2_ microspheres [5]. The *RL_max_* was −33.4 dB.

The non-homogeneous interface of ferrite is prone to polarization, and the charge distribution at the interface is uneven, making it prone to the polarization phenomenon. Zhang et al. utilized this characteristic to synthesize (Fe/FeO_x_)@C composites [70], which exhibit better absorption performance than Fe@C. At a thickness of 2 mm, the *EAB* of (Fe/FeO_x_)@C-2 increased by 26.3%, reaching 7.3 GHz (10.7–18.0 GHz). He et al. used N doping to regulate the electronic structure of carbon materials and prepared Fe_3_O_4_@C@Co/N-Doped C (FCCNC) composite materials, increasing dipole polarization. Figure 5a shows its loss mechanism. The conductive network generated by ZIF-67 carbonization, which connects Fe_3_O_4_ ferrite and carbon layers, enables dielectric-electromagnetic synergy and impedance matching, which is optimized and enhanced [71]. As shown in Figure 5c, the particles appear spherical in shape. The *RL_max_* of FCCNC reaches −66.39 dB, with a matching thickness of just 1.9 mm from Figure 5b.

The electromagnetic parameters and impedance matching characteristics of yolk-eggshell optimized materials are carbonized to form a carbon layer that combines with ferrite, thereby reducing reflection loss and improving absorption performance.

### 4.4. Non-Spherical Core-Shell Structure

**Figure 6:**
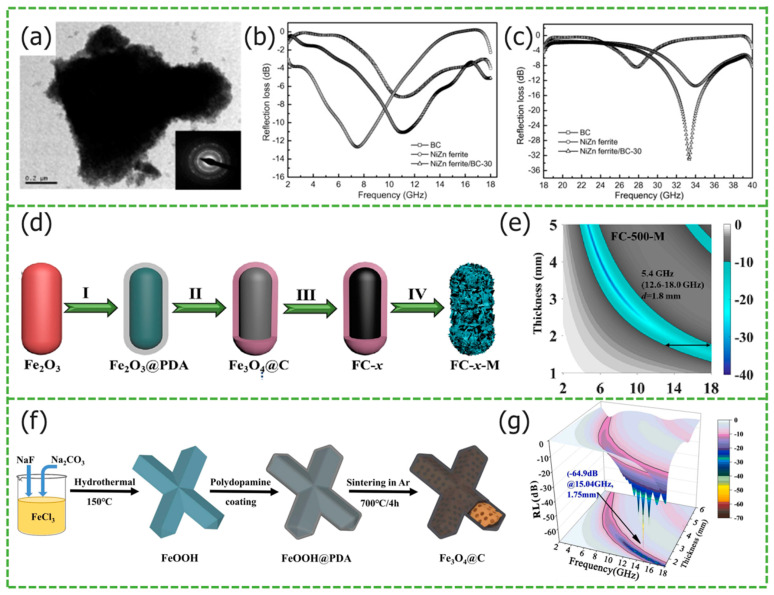
Graphical summary of irregular core–shell structure *MAMs*. (**a**) TEM photograph and the *RL* at (**b**) 2–18 GHz and (**c**) 18–40 GHz of NiZn ferrite/BC-30. Reproduced with permission [77] Copyright 2008, Elsevier Ltd. (**d**) Schematic of the preparation and (**e**) planar *RL* maps of FC-500-M. Reproduced with permission [76] Copyright 2023, Elsevier B.V. (**f**) Illustrated schematic for the preparation process and (**g**) 3D *RL* plots of Fe_3_O_4_ @C-60. Reproduced with permission [74] Copyright 2024, Elsevier B.V.

Besides the common spherical core–shell structures, there are also some non-spherical core–shell structures. Examples of these structures are spindle, ellipsoid, rod, nano-axis, and capsule. The size anisotropy influences interfacial polarization, leading to the unique properties of non-spherical core–shell structure *MAMs*. Xu et al. prepared Fe_3_O_4_@CuSiO_3_ nanoparticle composites utilizing a modified Stöber method [72]. The aspect ratio and dimensions of the elliptical structure influence interfacial scattering and polarization. Compared to spherical nanoparticles, the complex dielectric constant exhibits a double resonant peak in its real part, indicating a more intense interfacial polarization, and exhibiting anisotropy in its microwave absorption properties. The sea urchin-shaped external CuSiO_3_ shell wraps around the internal Fe_3_O_4_ magnetic core, creating a synergistic effect to help absorb electromagnetic waves, avoiding oxidation when exposed to air. At a sample thickness of 2 mm, the *RL_max_* is −30.8 dB and the *EAB* is 8 GHz. In the work of You et al., the synthesized γ-Fe_2_O_3_@C@α-MnO_2_ nano-axis composites [73] also exhibit anisotropy in terms of absorption performance. By controlling different ion concentration ratios, crystal growth direction can be guided, and a unique bipolar distribution cavity core–shell structure can be synthesized. Due to the high-temperature condensation properties of dopamine, an α-Fe_2_O_3_ ellipsoid is wrapped to form a carbon layer, optimizing impedance matching and the magnetic dielectric synergistic effect. When the sample thickness is 2mm, the *RL_max_* at 9.36 GHz is −45 dB, with an *EAB* of 3.89 GHz (7.66–11.55 GHz). Compared to traditional core–shell *MAMs*, it demonstrates a pronounced microwave absorption property. Lei et al. synthesized X-shaped Fe_3_O_4_@C composites using the hydrothermal surface coating sintering method, the preparation diagram is shown in Figure 6f [74]; these also have a similar adjustment mechanism, and their absorption performance is adjusted through the proportion of X-shaped dimensions. The sample exhibits a *RL_max_* of −64.92 dB at 15.04 GHz, with an *EAB* of 4.64 GHz (13.04–17.68 GHz) from Figure 6g. The matching thickness is only 1.75 mm, demonstrating outstanding microwave absorption performance.

By anchoring ferrite particles onto the surface of carbon fibers, Dai et al. developed core–shell structured C/Fe_3_O_4_ composites [75]. The many heterogeneous interfaces formed between graphite nanocrystals and amorphous carbon in carbon fibers lead to charge transfers and electron reconstruction at the interface. At the same time, new heterogeneous interfaces are formed between Fe_3_O_4_ particles and the surface of carbon fibers, ensuring C/Fe_3_O_4_ composite fibers have excellent absorption performance. The *RL_max_* at 17 GHz is −55.98 dB, with a matching thickness of only 1.0 mm. Liu et al. synthesized Fe/Fe_3_O_4_@C@MoS_2_ composites with a capsule-like structure [76]. The preparation diagram is shown in Figure 6d. Upon reduction of a small amount of Fe_3_O_4_ to Fe, the magnetic loss capability of the composite material is enhanced. For samples with a thickness of 1.8 mm, the *EAB* is 5.4 GHz in Figure 6e.

Biomass materials are widely available and possess a high carbon content. Wu et al. replaced Fe^3+^ with Ni^2+^ and Zn^2+^doping, and synthesized Ni_0.5_Zn_0.5_Fe_2_O_4_@bamboo charcoal (BC) core-shell nanocomposites utilizing the hydrothermal reaction technique [77]. The NiZn ferrite with an unsaturated coordination is encapsulated on the surface of BC from Figure 6a. The internal lattice defects act as ion relaxation polarization centers, accumulating a significant amount of charge, thereby enhancing polarization loss. The peak-to-peak amplitude of Ni_0.5_Zn_0.5_Fe_2_O_4_@ BC core-shell nanocomposites increase with increasing temperature, at temperatures ranging from 300 to 470 K. Due to the weakening of magnetic crystal anisotropy, the peak-to-peak linewidth decreases with increasing temperature. When the BC is present in a 30% volume, it exhibits superior microwave absorption properties in the Ka band. Within the broad frequency range of 2–40 GHz in Figure 6b,c, the *RL_max_* reaches −32.7 dB.

The unique shape of the non-spherical core-shell structure, with size anisotropy to regulate the absorption performance, makes it easier to synthesize high-performance *MAMs*. The electromagnetic testing of the above-mentioned ferrite *MAMs* shows that the core-shell structure has significant advantages in preparing *MAMs*.

## 5. Porous Structure

The microstructure of materials is distinctive, and through etching, unexpected porous structures can be produced. These gaps not only increase the contact area between the material and air, effectively reducing material quality and improving impedance matching, but also allow more electromagnetic waves to penetrate the material’s interior, enhancing the multiple *RL* of electromagnetic wave energy. Based on the formation mechanism of porous structure, they can be divided into two categories: one is the use of biomass carbon-based materials, with natural porous microstructures; the other type is prepared through reactive composting.

### 5.1. Biomass Porous Structure

Biomass-based carbon materials currently represent a research hotspot, possessing advantages such as high sustainability, low cost, novel structural designs, diverse synthesis methods, and high carbon content. The integration of biomass carbon with ferrite materials results in the preparation of porous *MAMs*. The perfect complementarity between their microwave-absorbing mechanisms ensures their outstanding performance, presenting a broad application potential in the field of microwave absorption. Biomass materials come from a wide range of sources, such as agricultural waste, fruit shells, and animal and plant materials. Typically, after undergoing high-temperature carbonization and activation processes, the microstructure of biomass carbon undergoes significant alterations. Biomass carbons produced by carbonization at 600 °C exhibit a higher density of disordered carbon layer defects, yet the porous structure retains its integrity relatively well. Ferrite after atomic doping replacement is chosen, and the composite material synthesized with it has stronger magnetic properties.

**Figure 7:**
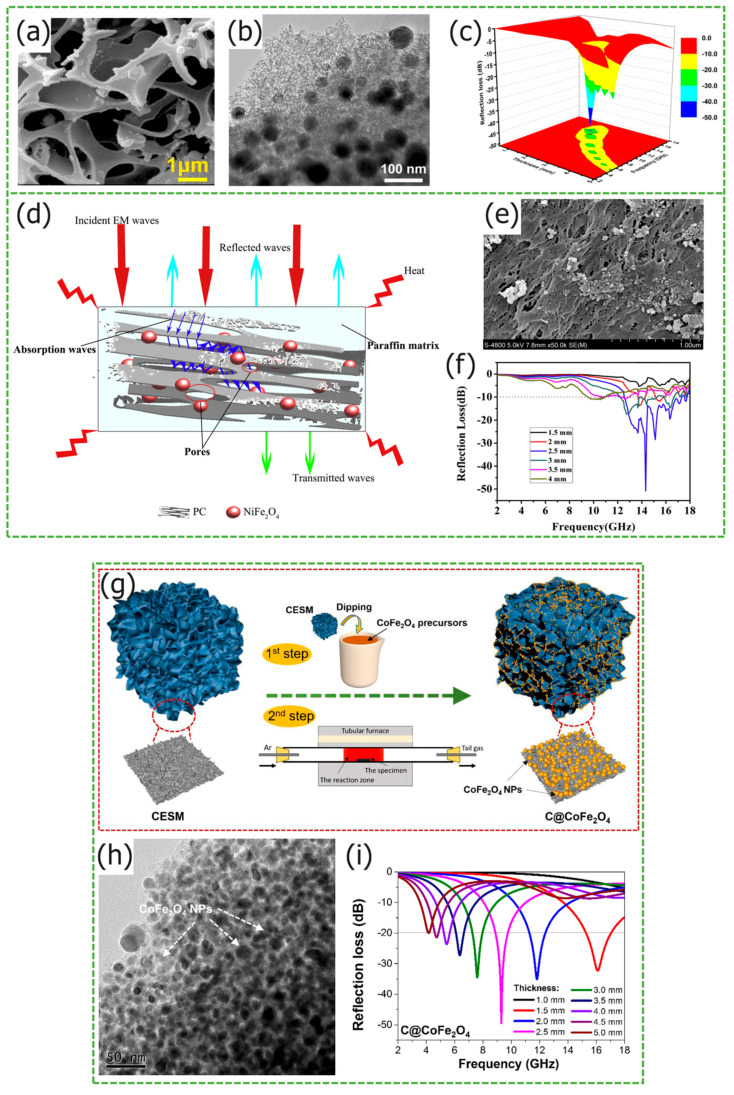
Graphical summary of biomass porous structure *MAMs*. (**a**) SEM image, (**b**) TEM image, and (**c**) 3D plots of *RL* of the MPC600. Reproduced with permission [81] Copyright 2018, American Chemical Society. (**d**) Schematic illustration of absorption mechanisms, (**e**) FESEM images, and (**f**) the *RL* curves of porous carbon@NiFe_2_O_4_. Reproduced with permission [78] Copyright 2019, Elsevier B.V. (**g**) Schematic illustration for preparation, (**h**) transmission electron microscope images, and (**i**) *RL* values of C/CoFe_2_O_4_. Reproduced with permission [80] Copyright 2019, Elsevier Ltd.

Wang et al. synthesized porous carbon @ NiFe_2_O_4_ composite materials using pomelo peel as a carbon source by replacing Fe^3+^ with Ni^2+^ [78]. From Figure 7e, layers of carbon are superimposed to form a 3D conductive network, with natural micropores distributed across the surface, enhancing the contact area with air, and optimizing impedance matching. The loss mechanism is shown in Figure 7d. When the composite material has a 2.5 mm thickness, its *RL_max_* at 14.3 GHz is −50.8 dB, and the corresponding *EAB* is 4.9 GHz (12.4–17.3 GHz) in Figure 7f. Corn stover is one of the major agricultural wastes, and recycling it is of great significance. Using corn straw and grapefruit peel as raw materials, Sun et al. replaced Fe^3+^ with Ni^2+^ and Co^2+^, prepared Ni_0.5_Co_0.5_Fe_2_O_4_/corn straw/grapefruit peel composites, which possess a 3D layered porous structure [79]. When the sample thickness is 3mm, the *RL_max_* is −43.95 dB, with an *EAB* of 4.81 GHz. Huang et al. used Co^2+^to replace Fe^3+^ and synthesized C@CoFe_2_O_4_ nanocomposites, using the eggshell membrane impregnation method, the preparation process is shown in Figure 7g [80]. Figure 7h shows that the CoFe_2_O_4_ particles are anchored onto the porous carbon matrix, resulting in a strong synergistic effect of electromagnetic interaction between the two, and enhancing the material’s microwave absorption performance, which was also confirmed by simulation experiments. When the sample is filled with 30% paraffin matrix, the *RL_max_* at 9.2 GHz is −49.6 dB in Figure 7i.

Compared to hydrothermal and solvothermal methods, simple solution impregnation and high-temperature carbonization treatment are more convenient. Wang et al. prepared porous carbon/Fe_3_O_4_@Fe composites by immersing sponge with Fe(NO_3_)_3_ solution and high-temperature carbonization [81]. From Figure 7a,b, it can be observed that the porous structure and ferrite particle distribution are distinct, respectively. Under the carbonization temperature of 600 °C, the sample exhibits a relatively high attenuation constant. When the thickness is as thin as 2 mm, the *EAB* range is between 13 and 18 GHz, with the *RL_max_* reaching −49.6 dB at 15.9 GHz in Figure 7c, highlighting outstanding microwave-absorbing capabilities. Fang et al. immersed cotton in an Fe(NO_3_)_3_.9H_2_O solution, subjecting it to carbonization treatments at various elevated temperatures, thereby preparing Fe_3_O_4_/C composites [82]. Fe_3_O_4_ nanoparticles of different sizes are dispersed on the hollow fiber wall of cotton, and the nanopores on the fiber surface help improve impedance matching, absorbing more electromagnetic waves. When prepared by carbonization at 600 °C, the sample with a thickness of 2.0 mm exhibits an *EAB* of 4.4 GHz (11.4–15.8 GHz), the *RL_max_* is only −22.1 dB, and the absorption performance is poor. In the work of Zhang et al., biochar/ferrite porous composites were prepared using bamboo as the carbon source. The pyrolysis temperature was set at 800 °C, and the sample matching thickness was 2.0 mm, the *RL_max_* reached −43.2 dB, and *EAB* was 14.2 GHz [83].

The preparation method for biomass porous *MAMs* is relatively simple. They have light weight and high absorption strength, are suitable for large-scale preparation, and have significant advantages compared to other structures.

### 5.2. Other Porous Structure

In addition to porous carbon materials, there are also porous microspheres, aerogels, porous foam, and other structures. The interior is filled with many pores, which not only reduce the mass and increase the specific surface area, but can also adhere to ferrite particles, optimize electromagnetic parameters, and enhance electromagnetic wave loss capacity.

**Figure 8:**
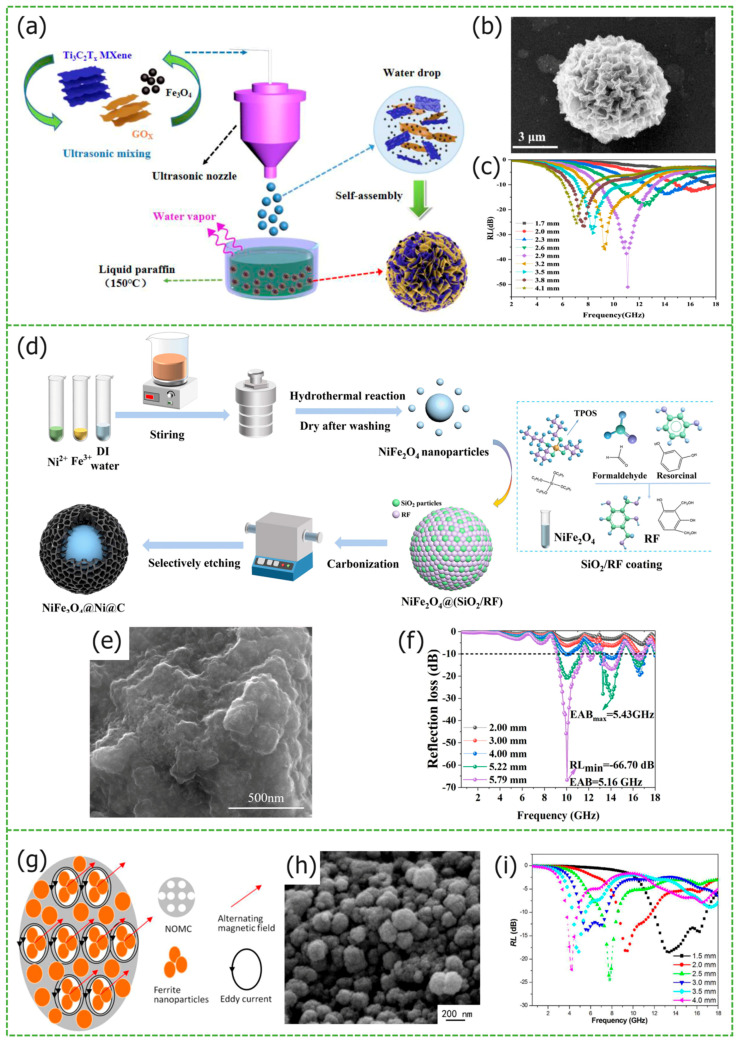
Graphical summary of other porous structure *MAMs*. (**a**) Experimental synthesized porous structure, (**b**) SEM images, and (**c**) the 2D *RL* plots of FMCM-3. Reproduced with permission [84] Copyright 2021, Elsevier Ltd. (**d**) Schematic diagram of preparation, (**e**) SEM images, and (**f**) *RL* of NiFe_2_O_4_@Ni@C-3. Reproduced with permission [85] Copyright 2022, Elsevier Inc. (**g**) Forming mechanism of eddy current, (**h**) SEM images, and (**i**) the *RL* curves of 40-F/NOMC. Reproduced with permission [89] Copyright 2020, Elsevier B.V.

Cui et al. synthesized RGO/MXene/Fe_3_O_4_ microspheres using the ultrasonic spray drying technique, the preparation process is shown in Figure 8a [84]. Under the influence of surface tension, droplets form into microspheres, which rapidly evaporate at high temperatures and adsorb Fe_3_O_4_ nanoparticles. These nanoparticles are distributed throughout the nanoplates assembled from RGO and MXene, leading to an irregular arrangement of nanoplates in Figure 8b that creates a porous structure, optimizing impedance matching. The synergistic effect of the three materials, while retaining their respective advantages, gives the microspheres enhanced microwave-absorbing properties. When the sample FMCM-3 is filled with 35% and has a thickness of 2.9 mm, its *RL_max_* at 11.1 GHz is −51.2 dB, with an *EAB* of 4.7 GHz from Figure 8c.

Liu et al. synthesized NiFe_2_O_4_@Ni@C composites, using a three-step process of a hydrothermal approach, in situ polymerization, and calcination, with porous and empty cavities inside the honeycomb structure [85], which promotes the electromagnetic wave energy loss, the preparation process is shown in Figure 8d. Figure 8e shows that porosity and cavities inside honeycomb structures. NiFe_2_O_4_ magnetic loss further enhances the absorption of microwaves, the *RL_max_* of the NiFe_2_O_4_@Ni@C sample is −66.70 dB, with an *EAB* of 5.16 GHz in Figure 8f.

Aerogel and porous foam have many interconnected pores, and a high specific surface area is conducive to increasing reaction sites and improving reaction efficiency. In the work of Xu et al., magnetic graphene foam@Fe_3_O_4_ composites were synthesized [86]. After the sample was subjected to acid treatment, it maintained a *RL_max_* of −49.4 dB at a thickness of 2.3 mm, with an *EAB* of 6.3 GHz (11.7–18.0 GHz). Fe_3_O_4_-modified carbon aerogel composites and SiO_2_/MXene/Fe_3_O_4_ aerogels were prepared by Ye et al. and He et al., respectively [87,88]. The porous structure optimizes the impedance matching, and the heterogeneous structure promotes the dielectric-magnetic synergy. The SiO_2_/MXene/Fe_3_O_4_ aerogel, with a thickness of merely 1 mm, exhibits an *EAB* reaching 8.8 GHz.

Similarly, using CoFe_2_O_4_ ferrite as the magnetic component, Shen et al. and Li et al. prepared CoFe_2_O_4_/ordered mesoporous carbon (NOMC) and CoFe_2_O_4_/carbon nanofiber (CNF) composites [89,90], respectively. In comparison to single components, the electromagnetic microwave absorption performance of composites is significantly enhanced. NOMC structure is shown in the Figure 8g,h, When the thickness of the 40-F/NOMC sample is a mere 1.5 mm, its *EAB* is 5.0 GHz (11.9–16.9 GHz) in Figure 8i. However, when CoFe_2_O_4_ doped with 20 wt% of quality, the CoFe_2_O_4_/CNF composites only exhibit an *EAB* of 3.6 GHz, with a matching thickness of 2.5 mm. This may be caused by poor impedance matching.

A porous structure with high porosity helps to increase the adsorption capacity of microspheres, facilitate their combination with other materials, and improve the absorption performance of ferrite composite materials.

## 6. Conclusions and Outlook

The development of *MAMs* with wide *EAB* values and a strong *RL* is the goal pursued by many researchers. Through continuous efforts, various structures of ferrite-based *MAM* have been explored, and are starting to be applied in the field of national defense and security. The above structures each have their own advantages. Using Fe_3_O_4_ ferrite as the matrix, a longitudinal comparative analysis was conducted on the absorption performance of composite materials. Table 1 shows absorption performance data of Fe_3_O_4_ MAMs with eight structures. Among them, the X-shaped Fe_3_O_4_@C composite material has the highest *RL_max_*, reaching −64.92 dB, but the *EAB* is relatively narrow. For the yolk-eggshell structure Fe_3_O_4_@SiO_2_, the *RL_max_* of the composite materials is only −36.5 dB, and the *EAB* covers 2–18 GHz, which means that the entire frequency range can absorb more than 90% of the electromagnetic microwave energy, demonstrating excellent performance. Compared with other structures, this indicates that the yolk-eggshell structure has significant advantages. The larger contact area between materials enhances interface polarization, and intensifies electron shift, electromagnetic microwave multiple reflections, and scattering of energy losses. Further research on the yolk-eggshell structure can be conducted, which also points out the direction for future research.

The integration of diverse materials can yield unique microstructures, while the multi-component synergistic optimization of the loss mechanism produces unexpected performances. This paper reviews the research progress on the structures of ferrite-based *MAMs*. Typically, ferrite is used to prepare composites with carbon-based compounds or MXene. The optimization of the microstructure of the synthesized composites faces numerous challenges.

In summary, the future structure of ferrite-based *MAM* can be approached from the following aspects:

(1) Design of new structures of ferrite *MAMs*. Based on the yolk–eggshell structure, the coating layer of the material is modified with pores to design a porous yolk–eggshell structure. For non-spherical core–shell structures, multi-layer hollow absorbing materials can be designed with different aspect ratios in different directions, which affect the synergistic loss mechanism between components. Determining how to synthesize these structures through experimental methods is currently a challenge that requires further research. The underlying mechanism of the influence of a material’s structure on its performance needs to be further explored to improve material stability.

(2) Optimization of the specific gravity of ferrite composites. An ideal *MAM* should be lightweight. Hence, reducing the specific gravity of ferrite composites is imperative. The reduction in specific gravity is a crucial method for altering the microstructure of materials. For instance, the test results demonstrated that aerogels and porous foam *MAMs* possess outstanding microwave absorption properties, effectively reducing their specific gravity. According to the density principle of composite materials, light weight should be achieved by introducing materials with lower density and combining them with ferrites. In composites, the distribution and morphology of dispersion have a significant impact on performance, and many research structures are currently randomly distributed. Determining how to control the dispersion of ferrites on the substrate, thereby achieving a controllable distribution, is one of the future directions.

(3) Development of multifunctional ferrite absorbing materials. Combining *MAMs* with other functional materials, such as catalysts and sensors, and integrating them with smart devices, can effectively improve the flexibility and intelligence level of the system in fields such as radio spectrum monitoring and antenna design, reduce the impact on the environment, improve electromagnetic compatibility and anti-interference ability, realize multifunctionality, and improve the application value. This integration can be achieved by applying absorbing materials to the external surface or internal structure of smart devices. The current difficulty lies in selecting suitable materials having a low cost, wide absorption bandwidth, and strong absorption ability, which restricts their application.

(4) Investigation of the degradation protection and technological scalability of ferrite *MAMs*. The degradation mechanism is a complex process that is influenced by environmental factors, such as high-temperature resistance, corrosion resistance, water and moisture resistance, and seismic protection. It is necessary to strengthen the corrosion resistance of materials to maintain good absorption performance in harsh environments and improve the service life of absorption materials. Realizing the large-scale manufacturing of *MAMs* is an important link for successful application. It is necessary to evaluate whether existing technologies can expand the manufacturing process scale, including the adaptability and feasibility of equipment, process flow, and raw materials, to enhance practical application.

## Figures and Tables

**Table 1:** Absorption performance data of Fe_3_O_4_ *MAMs* with eight structures.

Material	Structure Category	RLmax/dB	EAB/GHz	Matching Thickness/mm	Ref.
RGO@Fe_3_O_4_@PANI	sheet structure	−51.5	4.2	3.0	[13]
Fe_3_O_4_/RGO	Layered structure	−49.9	5.7	2.5	[31]
Fe_3_O_4_@C	Solid core-shell structure	−55.4	9.5	2.0	[52]
Fe_3_O_4_@PPy@RGO	hollow core-shell structure	−61.2	5.26	1.89	[64]
Fe_3_O_4_@SiO_2_	yolk-eggshell structure	−36.5	16	2.0	[68]
Fe_3_O_4_@C	non-spherical core-shell structure	−64.92	4.64	1.75	[74]
porouscarbon/Fe_3_O_4_@Fe	biomass porous structure	−49.6	5.0	2.0	[81]
Magnetic graphene foam@Fe_3_O_4_	other porous structures	−49.4	6.3	2.3	[86]

## Data Availability

Data are contained within the article.

## References

[1] Li X., You W., Wang L., Liu J., Wu Z., Pei K., Li Y., Che R. (2019). Self-Assembly-Magnetized MXene Avoid Dual-Agglomeration with Enhanced Interfaces for Strong Microwave Absorption through a Tunable Electromagnetic Property. ACS Appl. Mater. Interfaces.

[2] Zhang Y., Meng H., Shi Y., Zhang X., Liu C., Wang Y., Gong C., Zhang J. (2020). TiN/Ni/C ternary composites with expanded heterogeneous interfaces for efficient microwave absorption. Compos. Part B Eng..

[3] Fu Z., Lin C., Meng X. (2021). Three dimension Ni_0.5_Zn_0.5_Fe_2_O_4_/BaFe_12_O_19_@carbon composite for light weight, strong absorption and broadband microwave absorbents. Ceram. Int..

[4] Peng F., Meng F., Guo Y., Wang H., Huang F., Zhou Z. (2018). Intercalating Hybrids of Sandwich-like Fe_3_O_4_–Graphite: Synthesis and Their Synergistic Enhancement of Microwave Absorption. ACS Sustain. Chem. Eng..

[5] Meng F., Wang H., Wei, Chen Z., Li T., Li C., Xuan Y., Zhou Z. (2018). Generation of graphene-based aerogel microspheres for broadband and tunable high-performance microwave absorption by electrospinning-freeze drying process. Nano Res..

[6] Meng X., Dong S. (2019). Design and construction of lightweight C/Co heterojunction nanofibres for enhanced microwave absorption performance. J. Alloys Compd..

[7] Dong S., Lin C., Meng X. (2019). One-pot synthesis and microwave absorbing properties of ultrathin SrFe_12_O_19_ nanosheets. J. Alloys Compd..

[8] Li Y., Meng F., Mei Y., Wang H., Guo Y., Wang Y., Peng F., Huang F., Zhou Z. (2020). Electrospun generation of Ti_3_C_2_Tx MXene@graphene oxide hybrid aerogel microspheres for tunable high-performance microwave absorption. Chem. Eng. J..

[9] Xu C., Liu P., Wu Z., Zhang H., Zhang R., Zhang C., Wang L., Wang L., Yang B., Yang Z. (2022). Customizing Heterointerfaces in Multilevel Hollow Architecture Constructed by Magnetic Spindle Arrays Using the Polymerizing-Etching Strategy for Boosting Microwave Absorption. Adv. Sci..

[10] Quan B., Chen Y., Wang Y., Lu X., Guo T., Zhang M., Huang X. (2023). Synergistically enhanced flexibility, mechanical strength and microwave absorption performances of TPE-based hybrid films via thermally assisted homogeneous separation technology. Carbon.

[11] Lian Y., Han B., Liu D., Wang Y., Zhao H., Xu P., Han X., Du Y. (2020). Solvent-Free Synthesis of Ultrafine Tungsten Carbide Nanoparticles-Decorated Carbon Nanosheets for Microwave Absorption. Nano-Micro Lett..

[12] Huang L., Duan Y., Shi Y., Pang H., Zeng Q., Che R. (2022). Novel broadband electromagnetic-wave absorption metasurfaces composed of C-doped FeCoNiSiAl high-entropy-alloy ribbons with hierarchical nanostructures. Compos. Part B Eng..

[13] Wang H., Shi P., Rui M., Zhu A., Liu R., Zhang C. (2020). The green synthesis rGO/Fe_3_O_4_/PANI nanocomposites for enhanced electromagnetic waves absorption. Prog. Org. Coat..

[14] Min D. (2019). Enhanced Microwave Absorption Performance of Double-Layer Absorbers Containing BaFe_12_O_19_ Ferrite and Graphite Nanosheet Composites. J. Electron. Mater..

[15] Sandhya P.K., Jose J., Sreekala M.S., Padmanabhan M., Kalarikkal N., Thomas S. (2018). Reduced graphene oxide and ZnO decorated graphene for biomedical applications. Ceram. Int..

[16] Abazari S., Shamsipur A., Bakhsheshi-Rad H.R. (2022). Reduced graphene oxide (RGO) reinforced Mg biocomposites for use as orthopedic applications: Mechanical properties, cytocompatibility and antibacterial activity. J. Magnes. Alloys.

[17] Aldalbahi A., Samuel E., Alotaibi B.S., El-Hamshary H., Yoon S.S. (2021). Reduced graphene oxide supersonically sprayed on wearable fabric and decorated with iron oxide for supercapacitor applications. J. Mater. Sci. Technol..

[18] Li Q., Guo X., Zhang Y., Zhang W., Ge C., Zhao L., Wang X., Zhang H., Chen J., Wang Z. (2017). Porous graphene paper for supercapacitor applications. J. Mater. Sci. Technol..

[19] Sun J., Wang L., Yang Q., Shen Y., Zhang X. (2020). Preparation of copper-cobalt-nickel ferrite/graphene oxide/polyaniline composite and its applications in microwave absorption coating. Prog. Org. Coat..

[20] Deng Y., Li L., Wang L., Wu N., Jin H., Gao F., Zeng Z. (2024). Rare earth Ce-doped W-type barium ferrites for tunable electromagnetic waves absorption performance. Mater. Res. Bull..

[21] Ni Q., Sun L., Cao E., Hao W., Zhang Y., Ju L. (2020). Enhanced magnetic and dielectric properties of NiFe_2_O_4_ ferrite ceramics co-substituted by (Li^1+^, Zn^2+^ and La^3+^). Ceram. Int..

[22] Chireh M., Naseri M., Ghaedamini H. (2021). Enhanced microwave absorption performance of graphene/doped Li ferrite nanocomposites. Adv. Powder Technol..

[23] Shu R., Zhang G., Zhang J., Wang X., Wang M., Gan Y., Shi J., He J. (2018). Synthesis and high-performance microwave absorption of reduced graphene oxide/zinc ferrite hybrid nanocomposite. Mater. Lett..

[24] Li J., Zhou D., Liu W.-F., Su J.-Z., Fu M.-S. (2019). Novel and facile reduced graphene oxide anchored Ni-Co-Zn-Nd-ferrites composites for microwave absorption. Scr. Mater..

[25] Zhang N., Huang Y., Zong M., Ding X., Li S., Wang M. (2016). Coupling CoFe_2_O_4_ and SnS_2_ nanoparticles with reduced graphene oxide as a high-performance electromagnetic wave absorber. Ceram. Int..

[26] Wang Y., Fu Y., Wu X., Zhang W., Wang Q., Li J. (2017). Synthesis of hierarchical core-shell NiFe_2_O_4_@MnO_2_ composite microspheres decorated graphene nanosheet for enhanced microwave absorption performance. Ceram. Int..

[27] Yan J., Huang Y., Chen X., Wei C. (2016). Conducting polymers-NiFe_2_O_4_ coated on reduced graphene oxide sheets as electromagnetic (EM) wave absorption materials. Synth. Met..

[28] Gao X., Wang Y., Wang Q., Wu X., Zhang W., Zong M., Zhang L. (2019). Facile synthesis of a novel flower-like BiFeO_3_ microspheres/graphene with superior electromagnetic wave absorption performances. Ceram. Int..

[29] Liu P., Ng V.M.H., Yao Z., Zhou J., Lei Y., Yang Z., Kong L.B. (2017). Microwave absorption properties of double-layer absorbers based on Co_0.2_Ni_0.4_Zn_0.4_Fe_2_O_4_ ferrite and reduced graphene oxide composites. J. Alloys Compd..

[30] Zhao T., Jin W., Ji X., Yan H., Jiang Y., Dong Y., Yang Y., Dang A., Li H., Li T. (2017). Synthesis of sandwich microstructured expanded graphite/barium ferrite connected with carbon nanotube composite and its electromagnetic wave absorbing properties. J. Alloys Compd..

[31] Li W., Li B., Zhao Y., Wei X., Guo F. (2023). Facile synthesis of Fe_3_O_4_ nanoparticles/reduced graphene oxide sandwich composites for highly efficient microwave absorption. J. Colloid Interface Sci..

[32] Liu P., Huang Y., Yang Y., Yan J., Zhang X. (2016). Sandwich structures of graphene@Fe_3_O_4_@PANI decorated with TiO2 nanosheets for enhanced electromagnetic wave absorption properties. J. Alloys Compd..

[33] Lei Y., Yao Z., Li S., Zhou J., Haidry A.A., Liu P. (2020). Broadband high-performance electromagnetic wave absorption of Co-doped NiZn ferrite/polyaniline on MXenes. Ceram. Int..

[34] Li Y., Zhou X., Wang J., Deng Q., Li M., Du S., Han Y.-H., Lee J., Huang Q. (2017). Facile preparation of in situ coated Ti_3_C_2_Tx/Ni_0.5_Zn_0.5_Fe_2_O_4_ composites and their electromagnetic performance. RSC Adv..

[35] Guo S., Guan H., Li Y., Bao Y., Lei D., Zhao T., Zhong B., Li Z. (2021). Dual-loss Ti_3_C_2_Tx MXene/Ni_0.6_Zn_0.4_Fe_2_O_4_ heterogeneous nanocomposites for highly efficient electromagnetic wave absorption. J. Alloys Compd..

[36] Swapnalin J., Koneru B., Banerjee P., Natarajan S., Franco A. (2022). Multilayer intercalation: MXene/cobalt ferrite electromagnetic wave absorbing two-dimensional materials. J. Phys. Chem. Solids.

[37] Yang H., Dai J., Liu X., Lin Y., Wang J., Wang L., Wang F. (2017). Layered PVB/Ba_3_Co_2_Fe_24_O_41_/Ti_3_C_2_ Mxene composite: Enhanced electromagnetic wave absorption properties with high impedance match in a wide frequency range. Mater. Chem. Phys..

[38] Shi X., Wu Z., Liu Z., Lv J., Zi Z., Che R. (2022). Interface engineering in the hierarchical assembly of carbon-confined Fe_3_O_4_ nanospheres for enhanced microwave absorption. J. Mater. Chem. A.

[39] Liu J., Cheng J., Che R., Xu J., Liu M., Liu Z. (2012). Double-Shelled Yolk–Shell Microspheres with Fe_3_O_4_ Cores and SnO_2_ Double Shells as High-Performance Microwave Absorbers. J. Phys. Chem. C.

[40] Zhang N., Wang Y., Liu M., Cheng T., Xing Z., Li Z., Zhou W. (2024). Hollow Cu_2−x_S@NiFe Layered Double Hydroxide Core–Shell S-Scheme Heterojunctions with Broad-Spectrum Response and Enhanced Photothermal-Photocatalytic Performance. Small.

[41] Li L., Wang T., Zhang L., Su Z., Wang C., Wang R. (2012). Selected-control synthesis of monodisperse Fe_3_O_4_@C core-shell spheres, chains, and rings as high-performance anode materials for lithium-ion batteries. Chemistry.

[42] Kong F., Han Z., Tao S., Qian B. (2021). Core–shell structured SnSe@C microrod for Na-ion battery anode. J. Energy Chem..

[43] Chen Y., Luo J., Xu H., Hou X., Gong M., Yang C., Liu H., Wei X., Zhou L., Yin C. (2023). Core–Shell Structured Polyimide@γ-Al2O3 Nanofiber Separators for Lithium-Ion Batteries. ACS Appl. Energy Mater..

[44] Beka L.G., Li X., Wang X., Han C., Liu W. (2019). A hierarchical NiCo2S4 honeycomb/NiCo2S4 nanosheet core–shell structure for supercapacitor applications. RSC Adv..

[45] Zhu M., Luo Q., Lu C., Liu L. (2024). Yolk–shell Ni–Co bimetallic nitride/oxide heterostructures as high-performance electrode of all-solid-state supercapacitor. Appl. Organomet. Chem..

[46] Zhu M., Lu C., Ma Y., Yang Y. (2023). NiCo-glycolate-derived porous spherical NiCo_2_S_4_ for high-performance asymmetric supercapacitors. Appl. Organomet. Chem..

[47] Bhogal S., Kaur K., Malik A.K., Sonne C., Lee S.S., Kim K.-H. (2020). Core-shell structured molecularly imprinted materials for sensing applications. TrAC Trends Anal. Chem..

[48] Chen G., Wang Y., Xie R., Gong S. (2018). A review on core–shell structured unimolecular nanoparticles for biomedical applications. Adv. Drug Deliv. Rev..

[49] Zhu Z., Ouyang G., Yang G. (2013). The interface effect on the band offset of semiconductor nanocrystals with type-I core–shell structure. Phys. Chem. Chem. Phys..

[50] Zhou D., Li D., Zhou X., Xu W., Chen X., Liu D., Zhu Y., Song H. (2017). Semiconductor Plasmon Induced Up-Conversion Enhancement in mCu_2–x_S@SiO_2_@Y_2_O_3_:Yb^3+^/Er^3+^ Core–Shell Nanocomposites. ACS Appl. Mater. Interfaces.

[51] Guo C., Ding H., Xie M., Zhang H., Hong X., Sun L., Ding F. (2021). Multifunctional superamphiphobic fluorinated silica with a core-shell structure for anti-fouling and anti-corrosion applications. Colloids Surf. A Physicochem. Eng. Asp..

[52] Du Y., Liu W., Qiang R., Wang Y., Han X., Ma J., Xu P. (2014). Shell Thickness-Dependent Microwave Absorption of Core–Shell Fe_3_O_4_@C Composites. ACS Appl. Mater. Interfaces.

[53] Jia Z., Zhang J., Lv F., Hou Y., Liu J., Yu S., Liu S., Li L., Liu Y. (2023). Synthesis of Fe_3_O_4_@SiO_2_@C/Ni microspheres for enhanced electromagnetic wave absorption. J. Non-Cryst. Solids.

[54] Wang B., Ji Y., Mu C., Huo Y., Xiang J., Nie A., Xue T., Zhai K., Liu Z., Wen F. (2022). Well-controlled Core-shell structures based on Fe_3_O_4_ nanospheres coated by polyaniline for highly efficient microwave absorption. Appl. Surf. Sci..

[55] Zha L., Wei C., Liu J., Yang Y., Wu B., Zhang X., Wu J. (2024). The core-shell structure of nitrogen-doped carbon coated Fe_3_O_4_ decorated MXene for broadband and efficient microwave absorption. Ceram. Int..

[56] Shi X., Liu Z., Li X., You W., Shao Z., Che R. (2021). Enhanced dielectric polarization from disorder-engineered Fe_3_O_4_@black TiO_2_-x heterostructure for broadband microwave absorption. Chem. Eng. J..

[57] Chen X., Wang Y., Liu H., Jin S., Wu G. (2022). Interconnected magnetic carbon@NixCo_1-x_Fe_2_O_4_ nanospheres with core–shell structure: An efficient and thin electromagnetic wave absorber. J. Colloid Interface Sci..

[58] Ge Y., Li C., Waterhouse G.I.N., Zhang Z. (2021). ZnFe_2_O_4_@PDA@Polypyrrole composites with efficient electromagnetic wave absorption properties in the 18–40 GHz region. J. Mater. Sci..

[59] Zhu X., Dong Y., Pan F., Xiang Z., Liu Z., Deng B., Zhang X., Shi Z., Lu W. (2021). Covalent organic framework-derived hollow core-shell Fe/Fe_3_O_4_@porous carbon composites with corrosion resistance for lightweight and efficient microwave absorption. Compos. Commun..

[60] Chai L., Wang Y., Zhou N., Du Y., Zeng X., Zhou S., He Q., Wu G. (2021). In-situ growth of core-shell ZnFe_2_O_4_ @ porous hollow carbon microspheres as an efficient microwave absorber. J. Colloid Interface Sci..

[61] Zhang X., Zhang Y., He J., Li H., Bai Y., Gao S. (2023). ZnFe_2_O_4_ nanospheres decorated residual carbon from coal gasification fine slag as an ultra-thin microwave absorber. Fuel.

[62] Zhang Y., Li H., Gao S., Geng Y., Wu C. (2019). A study on the chemical state of carbon present in fine ash from gasification. Asia Pac. J. Chem. Eng..

[63] Gao S., Zhang Y., He J., Zhang X., Jiao F., Liu T., Li H., Wu C., Ma M. (2023). Coal gasification fine slag residual carbon decorated with hollow-spherical Fe_3_O_4_ nanoparticles for microwave absorption. Ceram. Int..

[64] Dong F., Dai B., Zhang H., Shi Y., Zhao R., Ding X., Wang H., Li T., Ma M., Ma Y. (2023). Fabrication of hierarchical reduced graphene oxide decorated with core-shell Fe_3_O_4_@polypyrrole heterostructures for excellent electromagnetic wave absorption. J. Colloid Interface Sci..

[65] Chen W., Zhu X., Liu Q., Fu M. (2017). Preparation of urchin-like strontium ferrites as microwave absorbing materials. Mater. Lett..

[66] Wu M., Rao L., Liu L., Li Y., Zhang Y., Ji Z., Ying G. (2023). Urchin-like Fe_3_O_4_@C hollow spheres with core–shell structure: Controllable synthesis and microwave absorption. J. Colloid Interface Sci..

[67] Zhang S., Qi Z., Zhao Y., Jiao Q., Ni X., Wang Y., Chang Y., Ding C. (2017). Core/shell structured composites of hollow spherical CoFe_2_O_4_ and CNTs as absorbing materials. J. Alloys Compd..

[68] Wang X., Liao M., Zhong Y., Zheng J.Y., Tian W., Zhai T., Zhi C., Ma Y., Yao J., Bando Y. (2012). ZnO hollow spheres with double-yolk egg structure for high-performance photocatalysts and photodetectors. Adv. Mater..

[69] Liu J., Xu J., Che R., Chen H., Liu M., Liu Z. (2013). Hierarchical Fe_3_O_4_@TiO_2_ yolk-shell microspheres with enhanced microwave-absorption properties. Chemistry.

[70] Zhang M., Wang L., Bao S., Song Z., Chen W., Jiang Z., Xie Z., Zheng L. (2023). A finite oxidation strategy for customizing heterogeneous interfaces to enhance magnetic loss ability and microwave absorption of Fe-cored carbon microcapsules. Nano Res..

[71] He P., Ma W., Xu J., Wang Y., Cui Z.K., Wei J., Zuo P., Liu X., Zhuang Q. (2023). Hierarchical and Orderly Surface Conductive Networks in Yolk–Shell Fe_3_O_4_@C@Co/N-Doped C Microspheres for Enhanced Microwave Absorption. Small.

[72] Xu J., Liu J., Che R., Liang C., Cao M., Li Y., Liu Z. (2014). Polarization enhancement of microwave absorption by increasing aspect ratio of ellipsoidal nanorattles with Fe_3_O_4_ cores and hierarchical CuSiO_3_ shells. Nanoscale.

[73] You W., Bi H., She W., Zhang Y., Che R. (2017). Dipolar-Distribution Cavity gamma-Fe_2_O_3_@C@alpha-MnO_2_ Nanospindle with Broadened Microwave Absorption Bandwidth by Chemically Etching. Small.

[74] Lei C.-X., Lin L.-F., Li S., Luo Q., Wang L.-S., Peng D.-L. (2024). Fabrication of porous X-shaped Fe_3_O_4_@C core-shell structures for tunable microwave absorption. J. Alloys Compd..

[75] Dai B., Qi Y., Song M., Zhang B., Wang N., Dai Y. (2022). Facile synthesis of core–shell structured C/Fe_3_O_4_ composite fiber electromagnetic wave absorbing materials with multiple loss mechanisms. J. Chem. Phys..

[76] Liu Y., Tian C., Wang F., Hu B., Xu P., Han X., Du Y. (2023). Dual-pathway optimization on microwave absorption characteristics of core–shell Fe_3_O_4_@C microcapsules: Composition regulation on magnetic core and MoS_2_ nanosheets growth on carbon shell. Chem. Eng. J..

[77] Wu K.H., Ting T.H., Liu C.I., Yang C.C., Hsu J.S. (2008). Electromagnetic and microwave absorbing properties of Ni_0.5_Zn_0.5_Fe_2_O_4_/bamboo charcoal core–shell nanocomposites. Compos. Sci. Technol..

[78] Wang Y., Gao X., Zhou H., Wu X., Zhang W., Wang Q., Luo C. (2019). Fabrication of biomass-derived carbon decorated with NiFe_2_O_4_ particles for broadband and strong microwave absorption. Powder Technol..

[79] Sun J., Chen J., Ge H., Yang Y., Wang H., Li N., Sun H. (2023). 3D hierarchical porous structure formed by CS/GP/Ni_0.5_Co_0.5_Fe_2_O_4_ for high-efficiency microwave absorption. Compos. Part A Appl. Sci. Manuf..

[80] Huang L., Li J., Wang Z., Li Y., He X., Yuan Y. (2019). Microwave absorption enhancement of porous C@CoFe_2_O_4_ nanocomposites derived from eggshell membrane. Carbon.

[81] Wang H., Meng F., Li J., Li T., Chen Z., Luo H., Zhou Z. (2018). Carbonized Design of Hierarchical Porous Carbon/Fe_3_O_4_@Fe Derived from Loofah Sponge to Achieve Tunable High-Performance Microwave Absorption. ACS Sustain. Chem. Eng..

[82] Fang Y., Xue W., Zhao R., Bao S., Wang W., Sun L., Chen L., Sun G., Chen B. (2020). Effect of nanoporosity on the electromagnetic wave absorption performance in a biomass-templated Fe_3_O_4_/C composite: A small-angle neutron scattering study. J. Mater. Chem. C.

[83] Zhang T., Zhao D., Wang L., Meng R., Zhao H., Zhou P., Xia L., Zhong B., Wang H., Wen G. (2020). A facile precursor pyrolysis route to bio-carbon/ferrite porous architecture with enhanced electromagnetic wave absorption in S-band. J. Alloys Compd..

[84] Cui Y., Yang K., Wang J., Shah T., Zhang Q., Zhang B. (2021). Preparation of pleated RGO/MXene/Fe_3_O_4_ microsphere and its absorption properties for electromagnetic wave. Carbon.

[85] Liu Z., Wang Y., Jia Z., Ling M., Yan Y., Chai L., Du H., Wu G. (2022). In situ constructed honeycomb-like NiFe_2_O_4_@Ni@C composites as efficient electromagnetic wave absorber. J. Colloid Interface Sci..

[86] Xu D., Xiong X., Chen P., Yu Q., Chu H., Yang S., Wang Q. (2019). Superior corrosion-resistant 3D porous magnetic graphene foam-ferrite nanocomposite with tunable electromagnetic wave absorption properties. J. Magn. Magn. Mater..

[87] He J., Li J., Zhang J., Yi P., Sun X., Han G., Li X., Zhang R., Liu X., Yu R. (2023). Metal ions-assisted construction of SiO_2_/MXene/Fe_3_O_4_ aerogel as multifunctional electromagnetic wave absorbing material. Carbon.

[88] Yang J., Ye Z., Wang K., Zhao Q., Li X. (2024). Nano-Fe_3_O_4_ decorated on carbon aerogel framework: Coupling microstructures synergistic effect for electromagnetic wave absorption. Adv. Powder Technol..

[89] Li Y., Yuan M., Liu H., Sun G. (2020). In situ synthesis of CoFe_2_O_4_ nanocrystals decorated in mesoporous carbon nanofibers with enhanced electromagnetic performance. J. Alloys Compd..

[90] Shen G., Mei B., Wu H., Wei H., Fang X., Xu Y. (2017). Microwave Electromagnetic and Absorption Properties of N-Doped Ordered Mesoporous Carbon Decorated with Ferrite Nanoparticles. J. Phys. Chem. C.

